# Protein-coated corrole nanoparticles for the treatment of prostate cancer cells

**DOI:** 10.1038/s41420-020-0288-x

**Published:** 2020-07-28

**Authors:** Matan Soll, Qiu-Cheng Chen, Benny Zhitomirsky, Punnajit P. Lim, John Termini, Harry B. Gray, Yehuda G. Assaraf, Zeev Gross

**Affiliations:** 1 Schulich Faculty of Chemistry, Technion–Israel Institute of Technology, 3200003 Haifa, Israel; 2 Department of Biology, The Fred Wyszkowski Cancer Research Laboratory, Technion–Israel Institute of Technology, 3200003 Haifa, Israel; 3 Department of Molecular Medicine, Beckman Research Institute of the City of Hope, Monrovia, CA 91010 USA; 4 Beckman Institute, California Institute of Technology, Pasadena, CA 91125 USA

**Keywords:** Drug development, Ion transport, Drug delivery, Calcium channels, Apoptosis

## Abstract

Development of novel therapeutic strategies to eradicate malignant tumors is of paramount importance in cancer research. In a recent study, we have introduced a facile protocol for the preparation of corrole-protein nanoparticles (NPs). These NPs consist of a corrole-core coated with protein. We now report that a novel lipophilic corrole, (**2**)Ga, delivered as human serum albumin (HSA)-coated NPs, displayed antineoplastic activity towards human prostate cancer DU-145 cells. Cryo-TEM analysis of these NPs revealed an average diameter of 50.2 ± 8.1 nm with a spherical architecture exhibiting low polydispersity. In vitro cellular uptake of (**2**)Ga/albumin NPs was attributable to rapid internalization of the corrole through ligand binding-dependent extracellular release and intercalation of the corrole cargo into the lipid bilayer of the plasma membrane. This finding is in contrast with a previously reported study on corrole-protein NPs that displayed cellular uptake via endocytosis. Investigation of the non-light-induced mechanism of action of (**2**)Ga suggested the induction of necrosis through plasma membrane destabilization, impairment of calcium homeostasis, lysosomal stress and rupture, as well as formation of reactive oxygen species (ROS). (**2**)Ga also exhibited potent light-induced cytotoxicity through ROS generation. These findings demonstrate a rapid cellular uptake of (**2**)Ga/protein NPs along with targeted induction of tumor cell necrosis.

## Introduction

Prostate cancer is the leading cause of cancer-related mortality in men worldwide^1^. Upon diagnosis, prostate cancer is typically organ-confined or only locally advanced^2^. Depending on multiple parameters including clinical stage and prostate specific antigen (PSA) levels, decisions are made whether to opt for active surveillance, radiotherapy, or prostatectomy^3^. Once the disease has spread outside the prostate, androgen deprivation therapy (ADT) via chemical castration is the treatment of choice^2^. Unfortunately, the response is only ephemeral and most patients will develop resistance to ADT, progressing towards castration-resistant prostate cancer (CRPC) within 18 to 36 months which may be ensued by metastatic castration-resistant prostate cancer (mCRPC)^2,4^. Despite recent advances in the therapy for mCRPC, this malignant disease remains incurable, although men suffering from this disease are living considerably longer these days^2,5^. Recent advances in this field of cancer research hold great promise for ablation of calcium homeostasis by utilizing a sarcoplasmic/endoplasmic reticulum calcium adenosine triphosphatase (SERCA) pump protein inhibitor in humans^6^.

The field of corroles, first discovered in 1964, experienced little progress until 1999 when the first efficient methodology for the synthesis of triarylcorroles was reported^7^. Later, work of great importance involved the design and synthesis of water-soluble corrole derivatives^8,9^, which paved the way for biomedical applications. Notably, water-soluble metal complexes were found to be promising drug candidates for the treatment of diverse medical disorders, ranging from conditions where cellular damage must be prevented (diabetes mellitus, heart, and neurodegenerative diseases) to the eradication of malignant cells^10,11^. The gallium(III) derivative of 5,10,15-tris(pentafluorophenyl)-2,17-bis(sulfonic acid)-corrole, (**1**)Ga, displayed potent cytotoxic effects toward cancer cells^12^, particularly when conjugated with a heregulin-modified protein that targeted the human epidermal growth factor receptor (HER). The corrole/protein complex aided in the detection of cancer cells in a nude mouse model bearing HER^+^ human tumors, and it killed tumor cells while sparing normal tissues^13^. In a more recent study, a gold(III) sulfonated corrole, ((**1**)Au), displayed elevated cytotoxic activity (relative to (**1**)Ga) toward various cancer cell lines, surpassing even cisplatin, a well-established and widely used chemotherapeutic^14^.

**Fig. 1:**
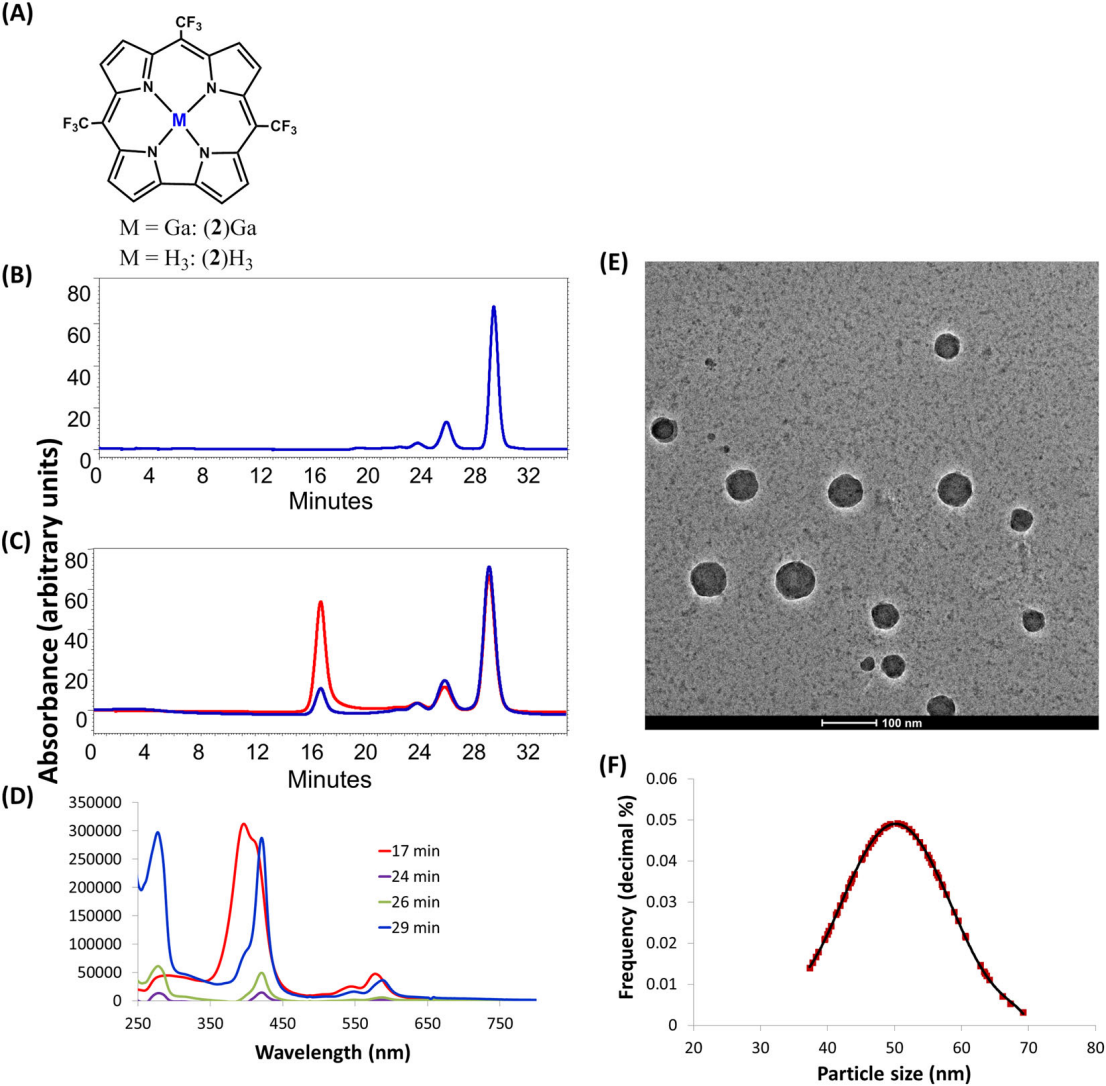
Characterization of protein-coated corrole nanoparticles. **a** Molecular structure of 5,10,15-tris(trifluoromethyl)corrole and its gallium(III) complex, (**2**)Ga. **b** HPLC chromatogram (Sephadex TM 200 10/300 GL column) for the detection of (**b**) HSA (only) and (**c**) HSA with (**2**)Ga in HSA/corrole NPs. HSA was detected at 280 nm (blue line) and corrole was detected at 422 nm (red line). **d** UV-Vis spectra recorded for HPLC-injected (**2**)Ga/HSA NPs at different retention times. **e** Representative Cryo-TEM imaging of (**2**)Ga/HSA NPs and (**f**) the size distribution of imaged particles.

Further investigation into the antineoplastic activity of metallocorroles revealed that the lipophilicity of corrole derivatives plays a central role in their activity toward malignant cells^15^. This finding led to the establishment of a facile procedure for the introduction of highly lipophilic corroles into biological media, consisting of corrole nanoparticles (NPs) coated with native proteins^15^. Independently, we also developed a novel one-pot synthesis of 5,10,15‐tris(trifluoromethyl)corrole and its Ga(III) derivative, (**2**)Ga (Fig. 1a), whose low molecular weight could be beneficial for medicinal applications^16,17^.

Here we report the antineoplastic activity of human serum albumin (HSA)-coated NPs of (**2**)Ga in the treatment of prostate cancer cells, particularly the well-documented androgen-independent DU-145 cell line, which displays moderate to high metastatic potential^18,19^. We observed fast cellular uptake and release of (**2**)Ga cargo from (**2**)Ga/HSA NPs into DU-145 cells in an extracellular, ligand binding-dependent manner. (**2**)Ga was found to induce necrosis by disrupting calcium homeostasis and by increasing the level of reactive oxygen species (ROS), and both proceeded more rapidly upon photoactivation. Importantly, our findings open the way for preclinical evaluation of these NPs as therapeutic agents against prostate cancer.

## Results

The formation of (**2**)Ga/HSA NPs was confirmed by utilizing our previously established protocols^15^. HPLC analysis (Fig. 1b–d) uncovered trends reminiscent of the reported NPs formed by 5,10,15-tris(pentafluorophenyl)corrole (TPFC) and its metal complexes. Analysis by size-exclusion chromatography revealed a fraction with an average retention time of 17 min, indicative of the formation of much larger particles than the later eluting albumin (Fig. 1b, c). In the HPLC-injected sample, (**2**)Ga is roughly equally distributed between the particle fraction at 17 min and the monomeric fraction at 29 min (Fig. 1b, c). UV-vis examination of the eluted fractions (Fig. 1d) supports this conclusion; the Soret band of protein-coated NPs is very broad (note also the λ_max_ = 280 nm due to HSA), while it is very sharp in the fraction eluting together with monomeric albumin. Assessment of particle size by the Nanosight NS300 system (Fig. S1) indicated a mean NP size of 69.2 ± 0.2 nm. Cryo-TEM analysis of (**2**)Ga/HSA NPs revealed the formation of particles that were morphologically distinct from tpfc-based NPs^15^. Particles were spherical with an average diameter of 50.2 ± 8.1 nm (Fig. 1e, f). Relative to tpfc-based NPs (~100 nm), (**2**)Ga formed smaller, more compact particles with lower polydispersity (Fig. S1).

**Fig. 2:**
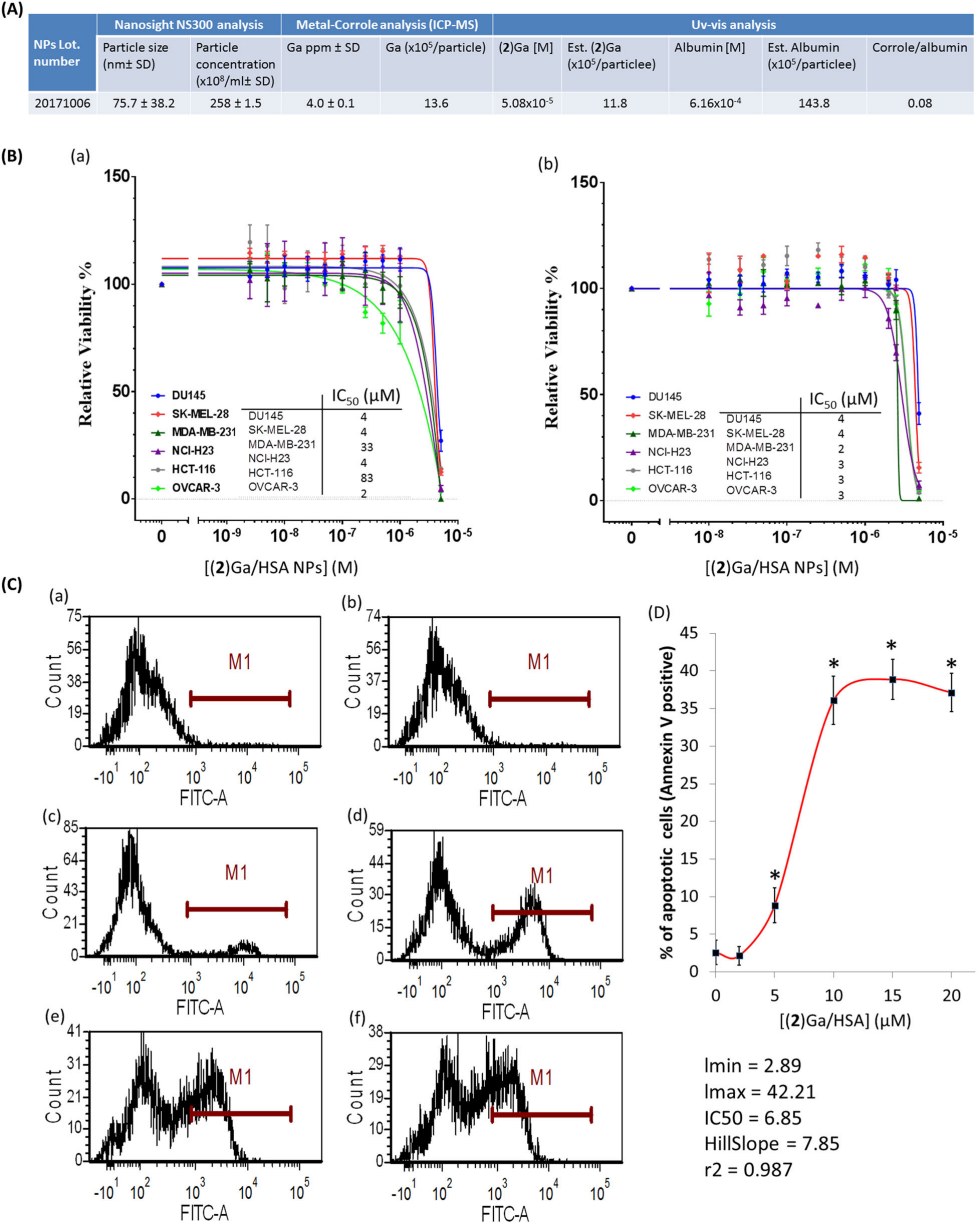
Cytotoxic and apoptotic effects of (2)Ga/HSA NPs in prostate cancer cells. **a** Size, particle concentration, corrole/protein ratios, and content parameters of (**2**)Ga/HSA NPs used in cytotoxicity assay. **b** Dose-response curves and IC_50_ values for a 48 and 72 h exposure (a and b, respectively) of human tumor cell lines to (**2**)Ga/HSA NPs were evaluated by a crystal violet staining assay. Data represent the mean ± SEM of two independent experiments. **c** AnnexinV-FITC staining of DU-145 cells incubated for 4 h with (a) HSA control and (**2**)Ga/HSA NPs at the following concentrations (b) 2 µM, (c) 5 µM, (d) 10 µM, (e) 15 µM, and (f) 20 µM. **d** Plotting of the gated cells (within M1) that are positive to AnnexinV-FITC (i.e., apoptotic cells) vs. (**2**)Ga concentration and determination of the IC_50_ value (IC_50_**=** 6.85 µM); data points represent the means ± SEM (*n***=** 3), *p**<** 0.001 vs. control untreated cells.

**Fig. 3:**
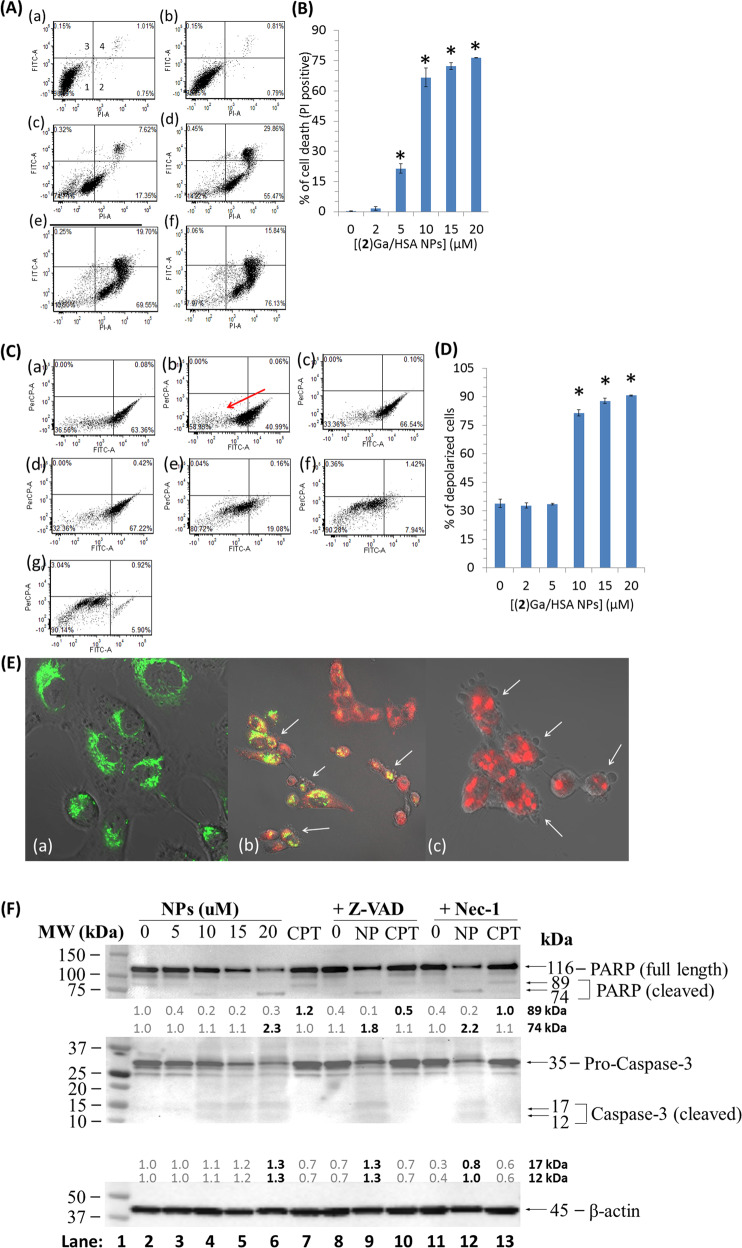
Detection of (2)Ga/HSA NPs uptake, cell death and disruption of mitochondrial membrane potential in prostate cancer cells. **a** AnnexinV-FITC staining of DU-145 cells incubated for 4 h with (a) HSA control and (**2**)Ga/HSA NPs at the following concentrations: (b) 2 µM, (c) 5 µM, (d) 10 µM, (e) 15 µM and (f) 20 µM. Propidium iodide (PI) was used as a nuclear counterstain to discriminate necrotic/dead cells. Panel 1 (Aa) represents the percentage of live cells (negative for both AnnexinV-FITC and PI). Panel 2 represents necrotic cells (PI positive). Panels 3 (AnnexinV-FITC positive) and 4 (AnnexinV-FITC and PI positive) represent early and late apoptotic/necrotic cells, respectively. **b** Summary of data sets depicting the percentage of tumor cell that were positively stained for necrosis (i.e., solely PI positive) under the same gating conditions from three distinct experiments; data points represent the means ± SEM (*n***=** 3), *p**<** 0.001 vs. control untreated cells. **c** Flow cytometric analysis of DU-145 cells treated with a mitochondrial depolarization reporter kit (MitoProbe^TM^ DiOC_2_(3)) after 4 h of incubation with: (a) HSA control; (b) CCCP, a protonophore and an uncoupling agent that blocks oxidative phosphorylation (positive control); and (c–g) (**2**)Ga/HSA NPs at 2, 5, 10, 15, and 20 µM, respectively. Note the red arrow pointing towards the left panel indicating the percentage of mitochondrial depolarization (obtained from CCCP-treated cells). **d** Summary of data sets depicting the percentage of tumor cells which stained positive for early mitochondrial depolarization (i.e., lower FITC fluorescence) under the same gating conditions from three distinct experiments; data points represent the means ± SEM (*n***=** 3), *p**<** 0.001 vs. control untreated cells. **e** Uptake of (**2**)Ga/HSA NPs by DU-145 prostate cancer cells after 4 h of incubation with: (a) HSA control + 30 min incubation (added to the wells just before imaging) with 200 nM MitoTracker green (MTG) followed by three washes; (**2**)Ga/HSA NPs at: (b) 5 µM, (c) 20 µM + 30 min incubation with 200 nM MTG and triple washes. Shown are superimposed images of conditions specific for detection of MTG (green fluorescence), (**2**)Ga (red fluorescence), and phase contrast images of the same field. Fluorescence was recorded using a ×20 objective and a LSM700 confocal microscope supported with Zen software. Samples were excited at 488 nm (3%) for MTG; and at 405 nm (10%) for (**2**)Ga detection. Representative images of 12 separate fields. White arrows point at possibly apoptotic cells (i.e., those displaying plasma membrane blebbing). **f** Western blot analysis of proteins isolated from cells pre-treated for 3 h with vehicle control, 50 µM of Z-VAD-FMK or Necrostatin-1. Following pretreatment, cells were incubated for 20 h with (**2**)Ga/HSA NP (NPs; 5, 10, 15, or 20 µM), NPs (15 µM), or camptothecin (CPT; 5 µM) without the removal of inhibitors. Band intensities were quantified using ImageLab software (Bio-rad) relative to vehicle control in lane 1. Representative results of two experiments are shown.

The cytotoxic activity of (**2**)Ga/HSA NPs (Fig. 2a) over a range of concentrations was first assessed against six cancer cell lines from the NCI-60 panel (Fig. 2b). IC_50_ values ranged from 2–83 µM after 24 h incubation to 2–4 µM after 48 h incubation, with greater cytotoxicity against MDA-MB-231 and HCT-116 cell lines upon 48 h exposure. The experiments were initially performed in the dark to minimize cytotoxicity due to corrole photoexcitation^10^. The initial assessment of (**2**)Ga/HSA NPs indicated that the DU-145 cell line responded more rapidly when compared to other cell lines upon treatment (plasma membrane blebbing and cell death were clearly visible by light microscopy within a few minutes). Since plasma membrane blebbing is considered a hallmark of apoptosis, we have used the Annexin V-FITC assay to detect apoptosis. Examination of DU-145 cells upon 4 h and 24 h incubation with (**2**)Ga/HSA NPs using this Annexin V-FITC apoptosis assay along with flow cytometry, confirmed the induction of cell death with IC_50_ values consistent with those obtained with crystal violet staining analysis (Fig. 2c, d, S2). After 4 h of incubation with (**2**)Ga/HSA NPs, there was a dose-dependent increase in the percentage of Annexin V-FITC fluorescent tumor cells, thus attaining 40% apoptotic cells at 15 µM NPs (Fig. 2c, d). Moreover, co-staining with propidium iodide (PI) showed cell death, attaining up to 85% dead cells upon 4 h treatment with 10 µM NPs, of which ~30% were positive for Annexin V-FITC, indicating late stage apoptosis or necrosis (Fig. 3a, b). Hence, a substantial component of cell death, apparently via necrosis, did not involve phosphatidylserine inversion. These results suggest that our NPs inflict a rapid tumor cell death predominantly via necrosis.

Mitochondrial depolarization, an established apoptotic/necrotic marker, was markedly detectable after 4 h of treatment with 10, 15, and 20 µM of (**2**)Ga/HSA NPs (Fig. 3c, panels e–g; Fig. 3d). Live microscopy imaging of the uptake of (**2**)Ga/HSA NPs by DU-145 cells employing MitoTracker green (MTG) staining after 4 h of incubation revealed both cell uptake of (**2**)Ga and mitochondrial depolarization, as MTG labels only actively respiring mitochondria (Fig. 3e, panels a, c). In cells exhibiting a positive mitochondrial MTG signal, i.e., those treated with 5 µM (**2**)Ga, co-localization of (**2**)Ga and MTG was clearly evidenced as yellow fluorescence (Fig. 3e, panel b). (**2**)Ga-treated cells apparently displayed apoptotic bodies and possibly plasma membrane blebbing visible by DIC microscopy, suggesting that some minor component of cell death presumably occurred via late apoptosis (Fig. 3e, panels b, c) on top of the predominant necrosis as shown below; moreover, cells treated with 20 µM (**2**)Ga formed large vesicular bodies (Fig. 3e, panel c). These results are consistent with our abovementioned observations indicating a rapid induction of cell death by (**2**)Ga/HSA NPs in prostate cancer DU-145 cells, even within minutes.

To characterize the molecular mechanism underlying the cell death induced by (**2**)Ga/HSA NPs, we have conducted experiments with the established pan-caspase inhibitor Z-VAD-FMK, as well as the RIP1 kinase (RIPK-1) inhibitor Necrostatin-1, which block apoptosis and necroptosis, respectively. Western blot analysis of both PARP cleavage and pro-caspase-3 activation by proteolytic cleavage, revealed intriguing results (Fig. 3f). (**2**)Ga/HSA NPs induced PARP cleavage and yielded a 74 kDa fragment which was slightly inhibited by Z-VAD-FMK but not with Necrostatin-1 (Fig. 3f, lanes 6, 9, 12). It should be noted that the 74 kDa PARP fragment is a hallmark of PARP cleavage by lysosomal cathepsin proteases released during necrosis^20,21^. Therefore, the slight inhibition of PARP cleavage to the 74 kDa fragment by Z-VAD-FMK could be explained by the non-specific activity of this inhibitor and its ability to also inhibit cathepsins B and H^22,23^. In contrast, 5 µM Camptothecin (CPT; Fig. 3f lanes 7, 10, 13), a cytotoxic antitumor agent which blocks topoisomerase I and induces apoptosis, brought about PARP cleavage and yielded the expected 89 kDa fragment—a hallmark of apoptosis. Expectedly, CPT-induced PARP cleavage was inhibited by Z-VAD-FMK, but not by Necrostatin-1 (Fig. 3f, lanes 10, 13). Surprisingly however, cleaved caspase-3 was not observed in CPT-treated cells (Fig. 3f, lanes 7, 10, 13). A potential explanation for this latter result is that the active form of caspase-3 is rapidly turned over, when compared to the pro-caspase-3 form in cancer cells^24^, making it difficult to observe the cleaved caspase-3, more so after the 20 h incubation period with CPT and (**2**)Ga/HSA NPs. In contrast, a dose-dependent increase in cleaved caspase-3 (Fig. 3f, 12 kDa and 17 kDa, lanes 4–6) could be detected after 20 h exposure to (**2**)Ga/HSA NPs. The NPs-induced cleavage of pro-caspase-3 was not inhibited by Z-VAD-FMK, but was blocked by Necrostatin-1 (Fig. 3f, lanes 9, 12). This indicates the role of the RIPK-1 pathway and caspase-8 in the observed activation of caspase-3^25^. Taken collectively, the apparently predominant mechanism of cell death induced by (**2**)Ga/HSA NPs largely proceeds via necrosis, accompanied by hallmarks of lysosomal membrane permeabilization and release of lysosomal cathepsin proteases. Future studies are warranted to reveal the hierarchy of the different pathways and their interconnection in the induction of this necrotic cell death.

**Fig. 4:**
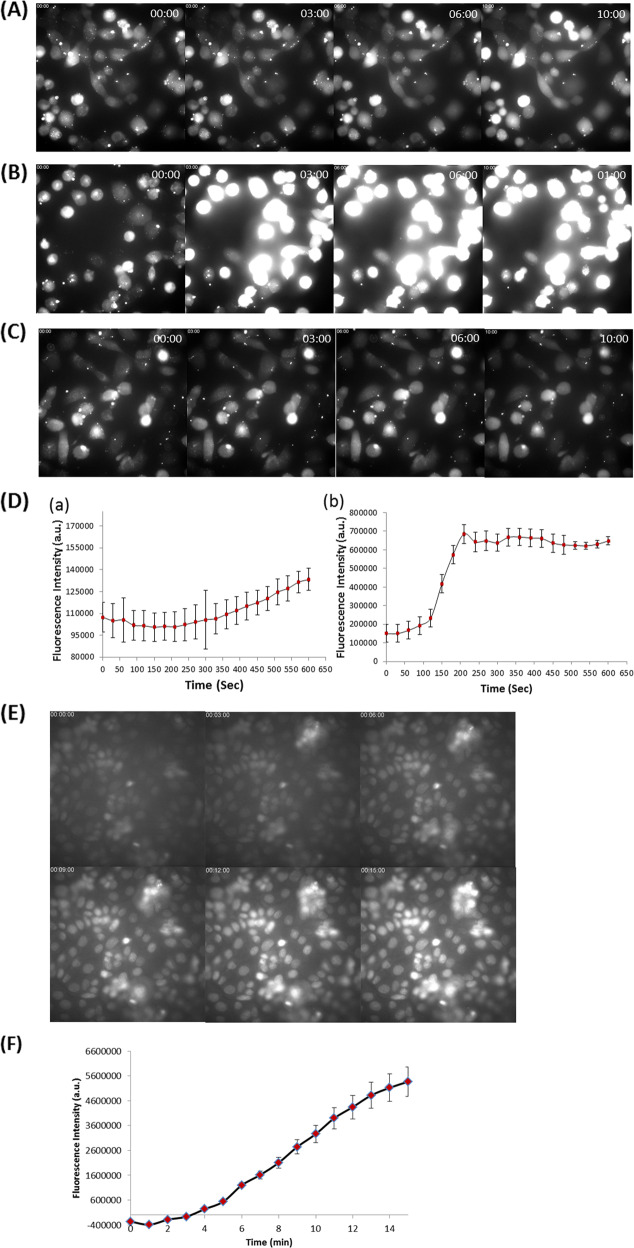
Kinetic study of cellular calcium flux and formation of ROS by (2)Ga/HSA NPs. Time-lapse fluorescence microscopy imaging of DU-145 cells (series ranging from time points *t***=** 0, 3, 6, and 10 min) incubated with the calcium indicator Fluo-8 AM (4 µM) for 30 min prior to triple washes and the addition of: (**a**) 2 µM (**2**)Ga/HSA NPs, (**b**) 20 µM of (**2**)Ga/HSA NPs, and (**c**) HSA control; (**d**) quantification of fluorescence emitted from each cell in 12 fields obtained from 2 µM (a) and 20 µM (b) of (**2**)Ga/HSA NPs. Data represent the mean ± SEM (*n***=** 6). (**e**) Time-lapse microscopy imaging of live DU-145 cells (series ranging from time points *t***=** 0, 3, 6, 9, 12, and 15 min) incubated with 10 µM of the ROS indicator CellROX green (accumulated mainly in the nucleus) for 30 min prior to triple washes and the addition of 20 µM of (**2**)Ga/HSA NPs- series of images taken at time intervals of 1 min ranging from *t***=** 0 (upper left corner) to *t***=** 15 min (lower right corner); time points are indicated in white on the upper left corner of each image; (**f**) quantification of fluorescence from the center of each cell nucleus in 12 different fields; data points are presented as mean ± SEM (*n***=** 6). Fluorescence was recorded using a ×20 objective and an IN Cell analyzer 2000 software (GE Healthcare). Samples were excited using a FITC filter for Fluo-8 AM or CellROX green detection. Images of 12 separate fields were obtained from 3 independent experiments.

The fast dynamics of cell death induction prompted us to assess cellular calcium flux as a potentiating factor in the (**2**)Ga cytotoxic mechanism. Monitoring intracellular calcium levels was performed using a calcium fluorescent indicator (Fluo-8 AM) and live time-lapse imaging in DU-145 cells (Fig. 4, Videos 1–3). Upon the addition of 20 µM (**2**)Ga/HSA NPs, a prominent sustained increase in cytosolic calcium content was observed 90 s after administration (Fig. 4b, d, panel b, Video 2). The intensity of fluorescence remained strong throughout the observation period, indicating elevated calcium release from intracellular compartments and/or influx from the extracellular milieu. The addition of 2 µM (**2**)Ga/HSA NPs led to similar results, but with a lower intracellular calcium signal. A minor elevation in the fluorescence from the detector was observed, between 7 and 10 min (Fig. 4a, d, panel a, Video 1). In contrast, no change in cellular calcium levels was observed in control cells incubated with HSA (Fig. 4c, Video 3). Repeating the assay with 2 µM (**2**)Ga/HSA in DU-145 cells with photoexcitation 30 s after NP addition, induced substantial elevation of intracellular calcium levels (Fig. S3a, Video S1). A single light pulse was sufficient to induce calcium levels comparable to that observed with 20 µM NPs under dark conditions, even with 10-fold lower NP concentration.

Calcium and ROS figure prominently as intracellular signaling molecules in regulating a multitude of cellular functions^26^. Since calcium and ROS display cross-talk between the cytosol and mitochondrial matrix, an experiment was designed to determine whether or not (**2**)Ga induces ROS production followed by intracellular calcium increase or vice versa.

The addition of CellROX green, a fluorescent ROS probe, revealed a strong elevation in cellular ROS levels after only 4 min following addition of (**2**)Ga/HSA NPs (20 µM; Fig. 4e, f). Since an intracellular calcium increase was observed 90 s after addition of (**2**)Ga/HSA NPs (*vide supra)*, it could have caused the induction of ROS^26^. The rapid intracellular calcium increase suggested the induction of phospholipid plasma membrane instability potentially involving nanopore disruption or formation of unstable regions in the bilayer. In accord with previous results, electronic excitation of (**2**)Ga in cells yielded much faster dynamics (less than 3 min) of ROS generation (Fig. S5, Video S4), while drug-free control cells did not (Video S5).

**Fig. 5:**
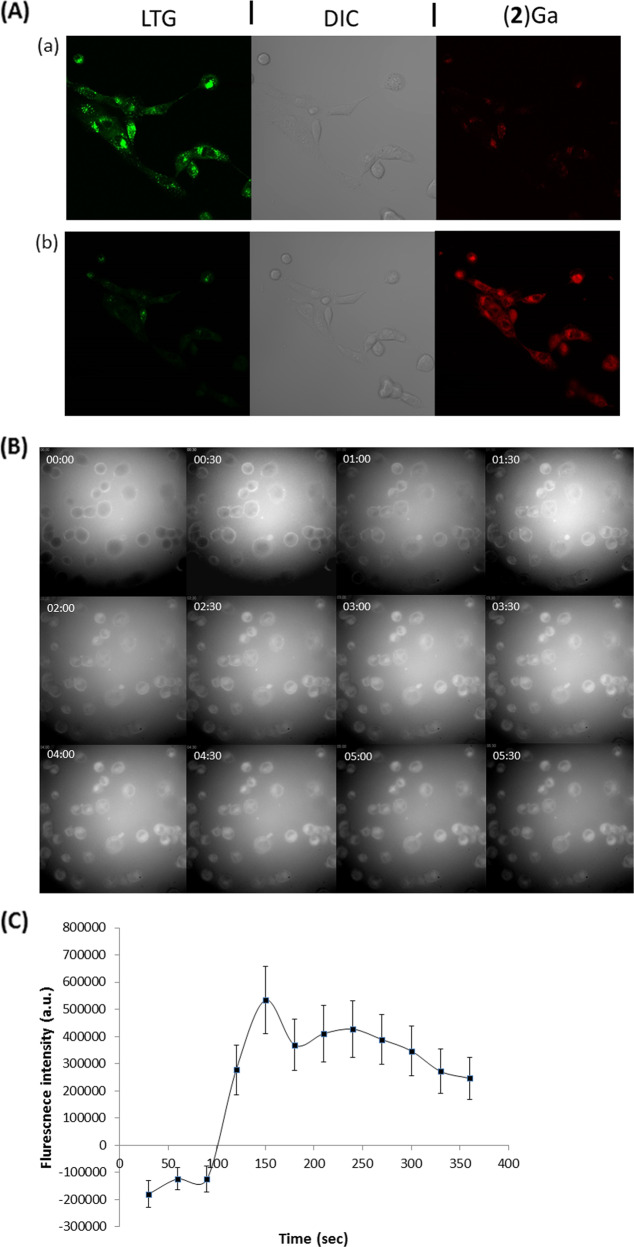
Kinetic of cellular internalization of (2)Ga/HSA NPs and disruption of lysosome in prostate cancer cells. **a** DU-145 cells: (a) after 30 min incubation with LysoTracker green (LTG) (150 nM); (b) same field after consecutive 10 min incubation with (**2**)Ga NPs (20 µM). Images were taken using conditions for specific detection of LTG (green fluorescence), (**2**)Ga (red fluorescence) and phase contrast images of the same field. Fluorescence was recorded at ×20 magnification using a LSM700 confocal microscope and Zen software. Samples were excited at 488 nm (3%) for LTG and at 405 nm (10%) for (**2**)Ga detection. Representative images of 12 separate fields and representative results of 3 independent experiments are shown. **b** Time-lapse microscopy imaging of (**2**)Ga/HSA NPs uptake in DU-145 cells: series of images taken at time intervals of 30 s ranging from *t***=** 0 (upper left corner) to *t***=** 6.5 min (lower right corner). The time point is indicated in white on the upper left corner of each image; (**c**) quantification of fluorescence from the center of each cell in 12 separate fields. Data points are presented as mean ± SEM (*n***=** 6). Fluorescence was recorded using a ×20 objective and an IN Cell analyzer 2000 software (GE Healthcare). Samples were excited using a CFP filter for (**2**)Ga detection. Representative images of 12 separate fields and representative results of 3 distinct repeats are presented.

To determine the extent of (**2**)Ga/HSA NPs localization within lysosomes, DU-145 cells were labeled with LysoTracker green (LTG), a lysosome-specific fluorescent dye. Within 10 min of treatment with (**2**)Ga/HSA NPs, the fluorescence was dramatically attenuated, implying the disruption of lysosomal structure (Fig. 5a, video 5, 6). This finding indicated destabilization of the outer cellular membrane leaflet and concomitant elevation of intracellular calcium levels. We conclude that membrane permeabilization/disruption must be exhibited by other cellular organelles. By following the uptake dynamics of (**2**)Ga/HSA NPs by DU-145 cells with time-lapse microscopy, fluorescence could be detected on the cell surfaces within the first few seconds (Fig. 5b, Video 7). Within 1 to 2 min of incubation, (**2**)Ga could be detected within cells, implying fast uptake dynamics with initial binding to the plasma membrane. On the other hand, no uptake of (**2**)H_3_/HSA NPs (the free base form of (**2**)Ga with the same quantum yield of fluorescence^16^, i.e., a comparable structural analog, Fig. 1a) could be detected within the time frame employed in the (**2**)Ga/HSA NPs imaging experiments (Fig. S6, Videos S6). Even after 1 h of incubation with (**2**)H_3_/HSA NPs, only several cells began to display a fluorescence signal in their plasma membrane from the fluorescent (**2**)H_3_ corrole (Fig. S7, Video S7), thereby indicating that the metal bound within the corrole macrocycle plays a role in corrole/HSA NPs uptake dynamics. The poor uptake of (**2**)H_3_ by DU-145 cells may account for the lack of changes in intracellular basal levels of calcium in cells treated with 20 µM (**2**)H_3_/HSA NPs (Fig. S3b, c, Video S2, S3). This is accentuated by the inability to obtain IC_50_ values for the (**2**)H_3_/HSA NPs in DU-145 cells after 24 h of incubation in dark conditions (Fig. S4). To address the possibility that these phenomena were solely due to monomeric corrole bound to HSA or vice versa (i.e., only due to the corrole NPs), we conducted an uptake experiment with virtually identical concentrations of isolated NPs and with albumin bound monomeric corrole isolated from the surfactant of NPs samples after centrifugation (×3 centrifugation isolation protocol). Both the isolated NPs and the monomeric albumin bound corroles (corresponding to peaks eluting at 17 and 29 min in Fig. 1) displayed uptake reminiscent of the non-separated formulation (i.e., initial membrane binding followed by fast partitioning into the cells) (Video 8, 9). We suggest that both monomeric bound corrole and corrole NPs display the same mechanism of uptake followed by induction of necrosis.

**Fig. 6:**
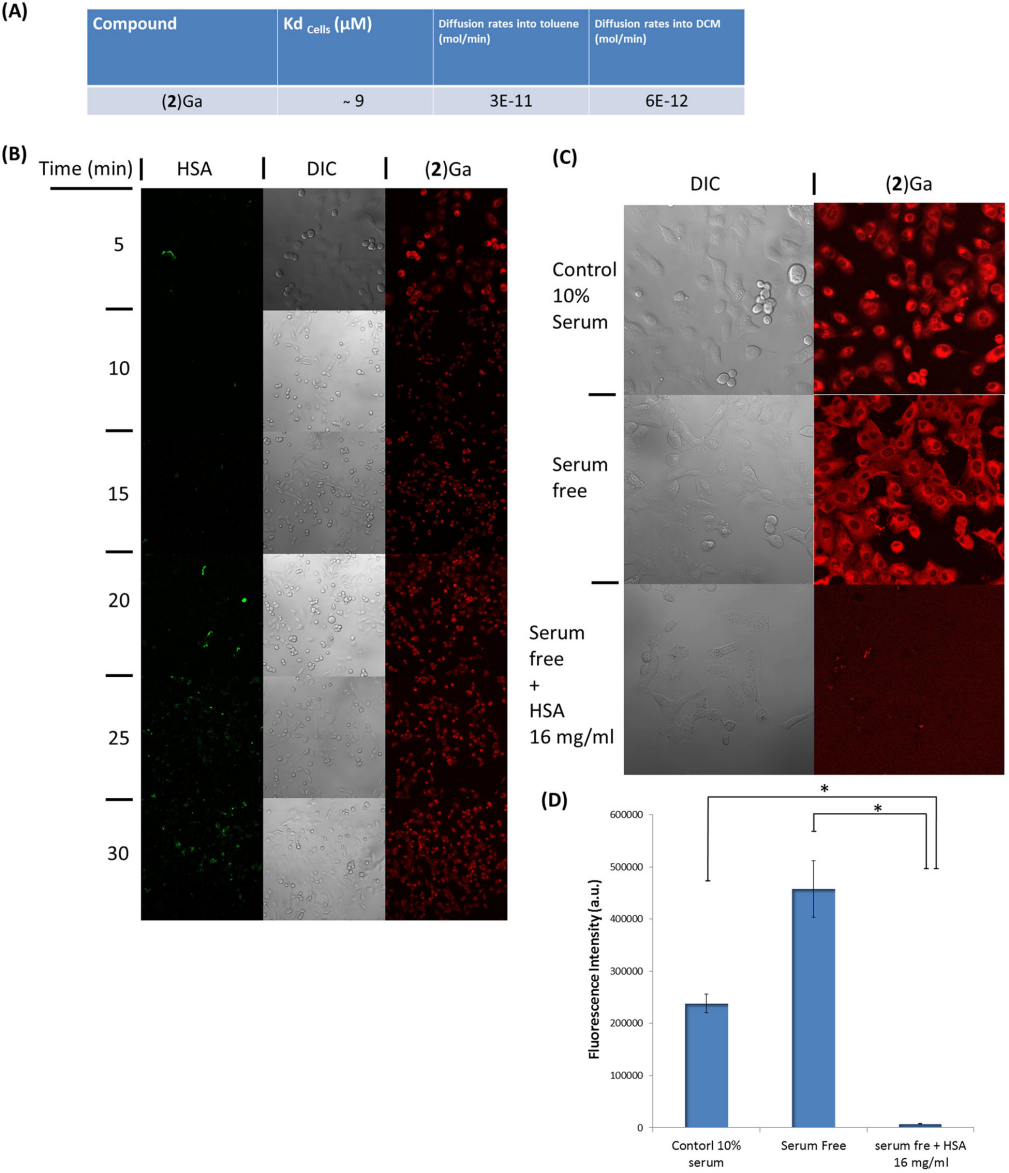
Characterization of the interaction of (2)Ga/HSA NPs with the plasma membrane of DU145 cells. **a** Table summarizing the binding affinity of (**2**)Ga/HSA NPs to DU-145 cells and (**2**)Ga diffusion rates from NPs into an organic phase. **b** Uptake dynamics of (**2**)Ga/HSA-FITC NPs (20 µM) measured by confocal imaging at time points of 5–30 min at 5 min intervals in the same treated well. **c** Uptake dynamics after incubation for 10 min with (**2**)Ga/HSA NPs (20 µM) followed by confocal microscopy imaging in the wells containing medium supplemented with 10% fetal calf serum, serum-free medium, and serum-free medium supplemented with 16 mg/mL HSA. **d** Quantification of fluorescence levels in each treatment group determined from 12 separate fields. Points are mean ± SEM (*n***=** 3), *p**<** 0.01. Shown are images of the conditions specific for the detection of HSA-FITC (green fluorescence), (**2**)Ga (red fluorescence), and phase contrast images of the same field. Fluorescence was recorded at ×10 magnification for (**b**) and ×20 magnification for (**c**) using a LSM700 confocal microscope and Zen software. Samples were excited at 488 nm (2%) for HSA; and at 405 nm (10%) for (**2**)Ga detection. Representative images of 12 independently derived photos obtained from 3 independent experiments.

To elucidate the role of HSA in cellular uptake of (**2**)Ga, the following questions were addressed: a) how crucial is plasma membrane binding for the uptake of (**2**)Ga/HSA NPs; and b) what is the role of HSA in the process of cellular internalization of (**2**)Ga (Fig. 6)? We first assessed the likelihood of simple diffusion through the plasma membrane, despite the fact that passive diffusion of (**2**)Ga is an unlikely mode of internalization considering the extremely slow diffusion rates of (**2**)Ga/HSA NPs dissolved in PBS into toluene or dichloromethane (DCM) (Fig. 6a, Fig. S8). As previously reported with other corroles^15^, extraction into DCM or toluene was not observed even upon vigorous stirring or shaking of the NPs in the PBS/DCM mixture (Video 10). Next, the uptake of (**2**)Ga/HSA NPs in regular media was examined by preparing NPs coated with HSA covalently modified with FITC (HSA-FITC). Upon incubation of DU-145 cells with (**2**)Ga/HSA-FITC NPs (20 µM), intracellular (**2**)Ga fluorescence could be detected within 5 min of incubation, while green fluorescence from FITC tagged HSA appeared after 15 min (Fig. 6b, Video 11- HSA-FITC, Video 12- (**2**)Ga). We conclude that (**2**)Ga/HSA NPs released their metallocorrole cargo upon extracellular binding to the plasma membrane. We considered the possibility that this result could have been due to exchange of HSA-FITC-NPs with non-fluorescent albumin from the media; however, upon repeating the experiment in serum free media, we obtained virtually identical results (Video S8- HSA-FITC channel, Video S9- (**2**)Ga channel).

To further describe the interaction of (**2**)Ga/HSA NPs with the plasma membrane, we used the Langmuir isotherm equation (Fig. S9) to determine the dissociation constant associated with (**2**)Ga/HSA NP-binding to DU-145 cells. Employing flow cytometry, we plotted (**2**)Ga emission maxima from cells incubated at 4 ^o^C with various concentrations of NPs (Fig. S9). From the fitted Langmuir plot, *K*_d_ is ~9 µM (Fig. S9). To confirm that the NPs remain bound to the cell surface, and that the cargo was not internalized into cells at 4^o^C, the experiments were repeated using an Amnis ImageStream®X Mark II apparatus for cell imaging during flow cytometry analysis. Fluorescence corresponding to the outer cell surface could be detected, but there was no fluorescent signal from the intracellular compartment (Fig. S10c). From the Langmuir plots, the estimated K_d_ using the Amnis system is ~7 µM (Fig. S10a, b). Since NP binding to the plasma membrane is in accord with the Langmuir model, including binding-site saturation, we suggest that there is specific binding to the cell surface. A simple competitive binding experiment was performed to validate this hypothesis: in this experiment, we added HSA to cells incubated in serum-free medium prior to adding (**2**)Ga/HSA NPs (20 µM) (Fig. 6c). Images obtained by confocal microscopy showed that cells incubated in serum-free medium displayed substantially stronger fluorescence from (**2**)Ga relative to cells in medium containing 10% serum (Fig. 6c, d), a finding that could be attributed to NP binding by albumin, as fetal calf serum contains substantial amounts of the protein. Cells that were incubated with excess HSA (16 mg/mL) in serum-free medium did not display uptake of (**2**)Ga after 10 min incubation (Fig. 6c, d), indicating that initial NP binding to the cell surface is essential for translocation of (**2**)Ga from an NP into the plasma membrane, which in turn is consistent with slow release rates of metallocorroles from NPs into organic phases (Fig. 6a, S8).

**Fig. 7:**
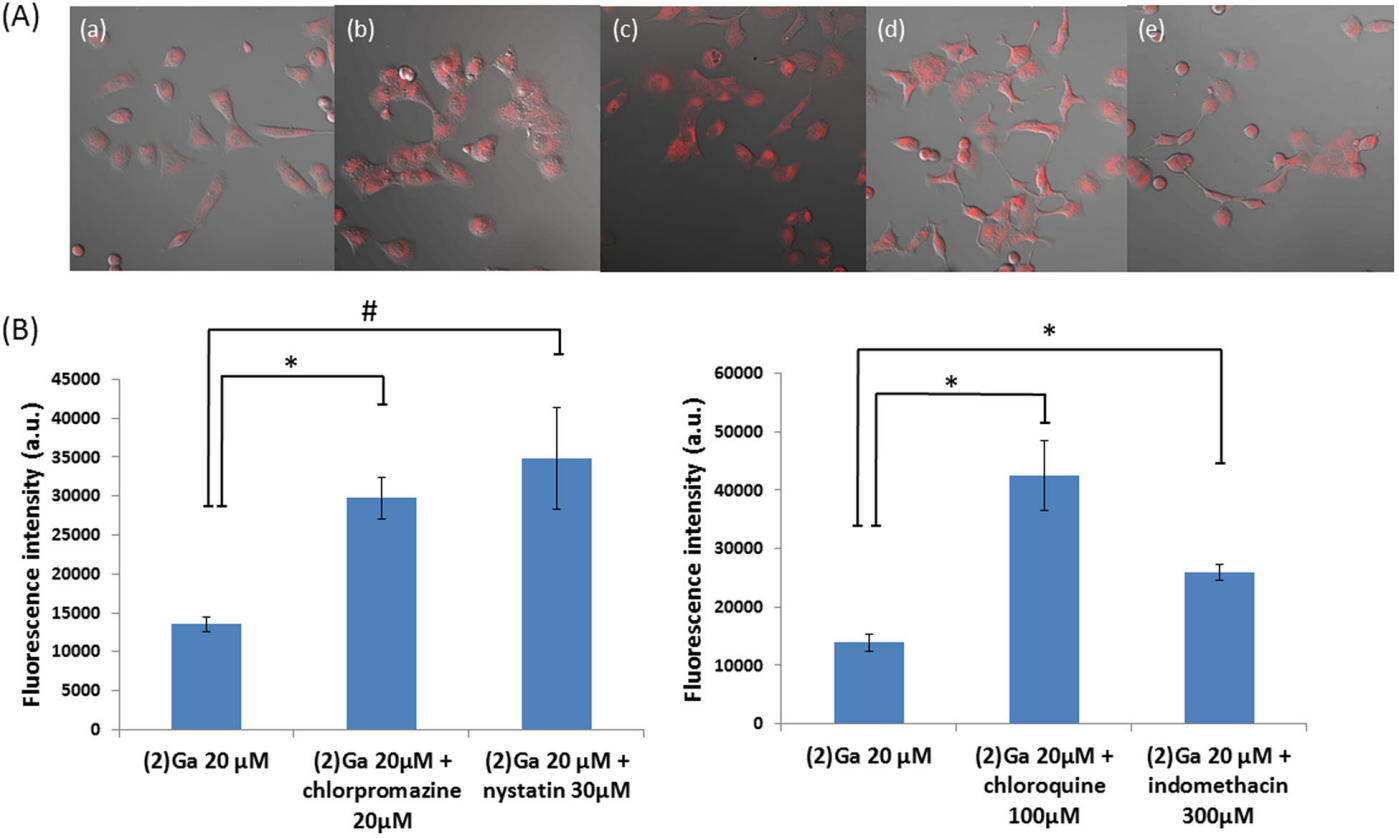
Effect of pharmacological endocytosis inhibitors on the uptake of (2)Ga/HSA NPs by DU-145 cells. **a** DU-145 cells were pre-incubated with: (a) vehicle, (b) chlorpromazine (20 µM), (c) nystatin (30 µM), (d) chloroquine (100 µM), and (e) indomethacin (300 µM) for 30 min; followed by cell incubation with (**2**)Ga/HSA NPs (20 µM) for 1 h and assessed by live confocal microscopy. Fluorescence was recorded at ×20 magnification using a LSM700 confocal microscope and Zen software. Samples were excited at 405 nm (10%) for (**2**)Ga detection. Representative images of 12 separate fields are presented. Shown are superimposed images with conditions specific for detection of (**2**)Ga (red fluorescence) and DIC images of the same fields; (**b**) quantification of fluorescence from each treated group measured from 12 separate fields. Points are shown as mean ± SEM (*n***=** 3), *p**<** 0.001, ^#^*p***<** 0.05. Results were obtained from 3 independent experiments.

To examine the potential role of endocytosis in (**2**)Ga uptake by DU-145 cells, the effect of well-established pharmacological inhibitors of both clathrin-mediated and caveolae-dependent endocytosis on NP uptake was determined. In particular, one clathrin inhibitor (chlorpromazine) has been shown specifically to inhibit albumin endocytosis^27–33^. Incubation of cells with both clathrin-mediated and caveolae-mediated endocytosis inhibitors for 30 min prior to addition of (**2**)Ga/HSA NPs was revealing: instead of inhibiting NP uptake, enhanced cellular uptake of (**2**)Ga was observed by confocal microscopy (Fig. 7). Chloroquine, chlorpromazine (clathrin-mediated endocytosis inhibitors), and nystatin (Caveolae-mediated endocytosis inhibitor), displayed substantial increases in cellular uptake of (**2**)Ga, whereas a more modest effect was found for indomethacin (caveolae-mediated inhibitor) (Fig. 7b). This surprising result could mean that there is reduced downregulation of extracellular target receptors, thereby increasing binding of (**2**)Ga/HSA NPs, which in turn would enhance uptake of (**2**)Ga by treated cells. The prominent difference between indomethacin and the other inhibitors could be explained in this way. Indomethacin does not only inhibit caveolae-mediated endocytosis, it also inhibits plasmalemmal vesicles from returning to the cell surface^30^, so the effect of overexpression of extracellular receptors (by blocking downregulation) would be mitigated by reduced fusion of plasmalemmal vesicles with the plasma membrane.

## Discussion

On the 29th of December 1959, the Nobel Laureate Richard P. Feynman gave a famous talk entitled “There’s Plenty of Room at the Bottom” at the Annual Meeting of the American Physical Society^34^. Extrapolating from known laws of physics; he envisioned a future dominated by the design and construction of nano-objects, atom by atom, molecule by molecule. If Feynman were alive today, he would say that NPs for drug delivery are prominent examples of the tiny objects he had in mind. Formulation of albumin-based NPs as controlled-release drug delivery systems, using advanced nanotechnology techniques such as desolvation, emulsification, thermal gelation, nano-spray drying, nab-technology, and self-assembly, has been widely investigated^35–41^. Examination of the literature reveals that the vast majority of NP formulations involve covalent or quaternary structural modifications of the albumin macromolecule. In contrast, we have developed a very efficient NP fabrication procedure in which lipophilic corroles and non-modified albumin assemble spontaneously^15^. In the current investigation, we employed our reproducible protocol to construct NPs containing the newly introduced (**2**)Ga, which, as it is much smaller than most corroles, likely would form smaller NPs with enhanced bioavailability (in accord with Lipinski’s rule of five)^42^. Indeed, we found that both the distribution of (**2**)Ga between monomeric HSA and NPs, as well as NP architecture were unlike other conjugated-particle properties. The (**2**)Ga/HSA NPs displayed remarkable homogeneity, with sizes ranging between 50–80 nm, well within the range (30–100 nm) that would allow accumulation in solid tumors^43^.

With a reproducible formulation in hand, the cytotoxicity of (**2**)Ga NPs was first tested by determining IC_50_ values in 6 different tumor cell lines. DU-145 was selected for in-depth investigations for the following reasons: (a) fast induction of cell death by (**2**)Ga in this cell line; (b) its relatively high metastatic potential^18,19^; (c) elevated levels of SPARC expression, the protein responsible for increased albumin uptake in metastatic prostate cancer cells^44,45^; and (d) its potential role as a preclinical in vitro model for mCRPC^46^.

The fast cellular uptake of (**2**)Ga appears to occur via facilitated diffusion, rather than through relatively slow endocytosis documented for NPs containing larger corroles^15^. Cumulative evidence suggests that (**2**)Ga/HSA NPs bind to a putative receptor on cell surfaces (K_d_ ~9 µM), thereby facilitating corrole redistribution from compact NPs into the plasma membrane. Blocking NP binding to the cell surface by excess serum albumin was sufficient to stop (**2**)Ga entry into DU-145 cells. Random diffusion of corrole from NPs into a lipid bilayer likely could not occur, owing to extremely slow release rates of (**2**)Ga into organic solvents. Surprisingly, (**2**)H_3_, in the form of (**2**)H_3_/HSA NPs, was not taken up by DU-145 membranes under virtually identical experimental conditions. We are puzzled by this different behavior, but we note that the poor uptake accords with the lack of cytotoxicity and impairment of calcium homeostasis displayed by (**2**)H_3_/HSA NPs. Inhibition of endocytosis by established inhibitors led to enhanced accumulation of (**2**)Ga, a finding that could be attributable to poor internalization of cell-surface-NP-bound receptors, which in turn would lead to an excess of receptors on the cell surface. These receptors would bind new NPs after disassembly of previously bound particles^47^.

The mechanism of action of (**2**)Ga under dark (light-free) conditions indicates impairment of calcium homeostasis, lysosomal stress, and eventually ROS formation, thereby inducing necrosis. This outcome is reflected in the fast kinetics (increased fluorescence) of the calcium probe Fluo-8 and the much slower kinetics of the ROS reporter CellROX green. The elevation of cytosolic calcium levels may induce production of ROS, which is likely mediated by cytosolic ROS-generating enzymatic sites (NADPH-oxidase, peroxisomes, cytochrome P450, xanthine oxidase, cyclooxygenase, and lipoxygenase)^26^. It also may stimulate ROS production via inhibition of mitochondrial electron transport^26^. The formation of large vesicular bodies containing (**2**)Ga (indicated by red fluorescence of imaged cells) and the loss of lysosomes inferred from the loss of LTG fluorescence may imply lysosomal stress, lysosomal membrane permeabilization and lysis as a necrosis mechanism; furthermore, the release of lysosomal cathepsin proteases (a necrosis hallmark) is due to loss of lysosomal membrane integrity and possible lysosome rupture. Further studies are warranted to delineate the pathways activated by (**2**)Ga, leading to this apparent necrotic cell death. The reason for calcium homeostasis impairment remains a mystery. Our initial results suggest that the focus should be on validating whether or not (**2**)Ga increases the fluidity of cellular membranes as recently shown for certain lipophilic compounds^48^. Cancer cells tend to exhibit a high density of saturated fatty acid-based phospholipids, rendering their membranes more rigid and much less fluid^49,50^: it follows that increasing membrane fluidity by (**2**)Ga may lead to altered transmembrane protein activity and signaling, which would be accompanied by uncontrolled calcium diffusion. Further research should shed light on the interaction of (**2**)Ga with cell membranes and its role in inducing cell death.

In 2016, Wilhelm and coworkers published a metadata survey of the NP-drug delivery literature^51^. This review revealed that NPs have made little impact in the field of nanomedicine. In the best cases in cancer therapy, for example, tumor accumulation did not exceed 10%. Given that an NP would need to undergo active endocytosis into malignant cells to release its drug cargo, it is not surprising that even approved (non-targeted) nanomedicines such as Abraxane and Doxil are not very effective^52–54^; indeed, their approval is rather due to improved toxicological profiles (not improved therapeutic activity)^55^. Although NP accumulation in malignant tumors is low, investigations of their uptake kinetics, drug loading and mode of drug release likely will greatly influence therapeutic profiles. The extracellular, binding-dependent, fast release of (**2**)Ga/HSA NPs may prove to be of importance in understanding drug release dynamics and its impact in nanomedicine. Furthermore, our finding of rapid induction of necrotic cell death by (**2**)Ga likely could influence the design of future nanomedicines.

## Methods

### Chemicals and reagents

EMEM growth medium, fetal bovine serum, penicillin-streptomycin, and supplements were purchased from Biological Industries (Beit-Haémek, Israel). MitoTracker Green (MTG), LysoTracker green (LTG), and ER-tracker green (ETG) were obtained from Life Technologies, Rhenium (Jerusalem, Israel). Z-VAD-FMK (#S7023) and necrostatin-1 (#S8037) were purchased from Selleck Chemicals (Houston, TX). The materials used for synthesis and work-up procedures were purchased from Sigma Aldrich, Merck, Fluka, and Frutarom and used as received unless otherwise stated. Deuterated solvents (Sigma Aldrich isotopes products) with a 99.5% minimum deuteration were used as received. Silica gel for column chromatography (Silica Gel 60, 63–200 µm mesh) was obtained from E. Merck Ltd. Pyrrole was run through a short basic alumina column and aldehydes were purified by vacuum distillation before use. Anti-caspase-3 polyclonal (CST#9662), polyclonal anti-PARP (CST#9542), and anti-β-actin monoclonal (CST#4970) antibodies were purchased from Cell Signaling Technology (Danvers, MA). Anti-Cleaved Caspase-3 monoclonal antibody (#ab32042) and anti-rabbit IgG H&L HRP-conjugated (#ab6721) antibodies were from Abcam (Cambridge, MA).

### Synthetic procedures

**(2)**Ga used for human serum albumin coated NPs was prepared using a previously reported procedure^16^.

#### Formulation of albumin/corrole nanoparticles

A solution of 400 µL of 1 mM corrole dissolved in DMSO was added dropwise (0.04 mL/min) to a 3.6 mL of 100 µM BSA or HSA in PBS, pH 7.2, and stirred at 500 rpm in a 5 °C water bath. Solutions were incubated for 30 min at 5 °C and transferred to dialysis tubing for 24 h dialysis in a 1 L PBS solution. Dialysis tubes were purchased from Spectrum labs, cat. No. 3787-D20; type: RC; MWCO: 12-14000. Dialysis tubing was treated vigorously with EDTA: tubing was immersed in 1 L of 2% sodium bicarbonate/1 mM EDTA solution in a 2 L glass beaker. Tubing was rinsed thoroughly with ddH_2_O (sterile ultra-pure water) and submerged completely in 50% ethanol/1 mM EDTA and stored at 4 °C. Tubing was rinsed thoroughly before use.

#### DLS and nanosight NS300 systems

Samples of NPs were diluted 10,000-fold in PBS. They were then measured using the Nanosight NS300 or the PSS Nicomp 380 DLS-ZLS Analyzer in accordance with the manufacturer’s instructions.

### 1.3.5 Cryo-TEM analysis

Samples of NPs were prepared to be at a mass percentage of 1% particles in PBS and the Cryo-TEM specimens were prepared in a controlled environment vitrification system (CEVS). Cryogenic transmission electron microscopy (Cryo-TEM) imaging was performed using a FEI Talos 200C, FEG-equipped Cryo-dedicated high-resolution transmission electron microscope (TEM and STEM), operated at an accelerating voltage of 120 kV. Specimens were transferred into an Oxford CT-3500 Cryo-holder (Philips) or a Gatan 626DH (FEI) Cryo-holder, and equilibrated below −178 °C. Specimens were examined using a low-dose imaging procedure to minimize electron-beam radiation damage. Images were recorded digitally by a Gatan Multiscan 791 cooled CCD camera (Philips CM 120), or a Gatan US 1000 high-resolution CCD camera (Tecnai T12 G2), using DigitalMicrograph software.

### HPLC

HPLC analysis was performed using a MERCK HITACHI HPLC system with a diode array detector supported with HPLC Chromaster Driver for Waters® Empower™3 Software. Ten microliter of each sample were injected using the auto-sampler. Size exclusion chromatography was performed using a sephadexTM 200 10/300 GL column, with 0.5 mL/min elution rate using sterile PBS (Sigma, sterile-filtered, isotonic, pH 7.2) as eluent.

### Human cancer cell lines

Six cell lines from the NCI-60 cell panel representing six distinct tumor types were used in this study: DU-145, prostate carcinoma; MDA-MB-231, breast adenocarcinoma; NCI-H23, lung adenocarcinoma; HCT-116, colorectal carcinoma; SK-MEL-28, malignant melanoma; and OVCAR-3, ovarian adenocarcinoma. Cells, except DU-145, were grown in RPMI-1640 medium (Mediatech) containing 2 mM L-glutamine, supplemented with 10% FBS (Omega Scientific). DU-145 (human prostate cancer), which was extensively used in this study, was grown in EMEM cell culture medium (ATCC) containing 2 mM L-glutamine, supplemented with 10% fetal bovine serum (FBS, Biological Industries), and maintained at 37 °C under 5% CO_2_ in a humidified incubator. Cell lines were maintained up to 10 passages.

### Crystal Violet growth inhibition assay

Tumor cells (DU-145, MDA-MB-231, SK-MEL-28, NCI-H23, HCT-116, and OVCAR-3) were seeded in 96-well microtiter plates (5 × 10^3^ cells per well; 90 μL per well) 24 h before the addition of the assigned NPs. At the time of drug treatment, stock solutions of compounds were diluted to 10-fold the desired final test concentrations with RPMI-1640 medium. Aliquots of 10 μL of these diluted solutions were added to the appropriate microtiter wells containing 90 μL of growth medium, resulting in the required final drug concentrations (eight concentrations per compound, ranging from 0.3 to 600 μM). The final concentration of DMSO (the compound required DMSO for solvation) in test culture was <1%. All cells were incubated in the dark throughout the 24–72 h exposure period and did not receive prolonged exposure to light. Following 24–72 h of exposure at 37 °C, cell viability was determined using the crystal violet assay (Promega/Sigma Aldrich/Sigma Aldrich) according to the manufacturer’s instructions. Absorbance was measured using a microplate reader (Synergy 4; Biotek Instruments) at 595 nm. Experiments were performed in triplicates and each dose-response curve represents the mean of three or more independent experiments. Spectrophotometric data were analyzed by sigmoid dose-response, nonlinear regression analysis; the IC_50_ values and associated SEM were calculated using GraphPad Prism 6 (GraphPad Software) or with Microsoft excel software.

### Flow cytometry analysis

DU-145 cells were seeded in 6-well plates (5 × 10^4^ cells/mL; 2.7 mL per well) 24 h before the addition of assigned compounds/albumin NPs. At the time of drug treatment, stock solutions of compounds were diluted to 10-fold the desired final test concentrations in EMEM medium. Aliquots of 300 μL of these diluted drug solutions were added to the appropriate microtiter wells containing 2.7 mL of growth medium, resulting in the required final drug concentrations. The final concentration of DMSO (given that the compound required DMSO for solvation) in test culture was <1%. All cells were incubated in the dark throughout the 24–72 h exposure period and did not receive prolonged exposure to light. Following 24–72 h of exposure at 37 °C, cell apoptosis/cell cycle/mitochondrial depolarization were determined with Annexin V-FITC kit (Sigma Aldrich)/ MitoProbe™ DiOC2(3) Assay Kit (Life Technologies, Rhenium, Jerusalem, Israel), respectively, according to the manufacturer’s instructions. Samples were taken for measurement using a BD LSR-II Analyzer supported with BD FACSDiVaTM Software Version 6.1 for data analysis. Corrole fluorescence in binding analysis was measured using appropriate filters for excitation (405 nm, DAPI) and emission (<590 nm, Ds-RED).

### Live cell imaging

DU-145 cells were seeded in 24-well plates containing a glass optic bottom (5 × 10^4^ cells/mL; 0.9 mL per well) 24 h before the addition of the assigned NPs. At the time of drug treatment, stock solutions of compounds were diluted to 10-fold the desired final test concentrations in EMEM medium. Aliquots of 100 μL of these diluted solutions were added to the appropriate microtiter wells containing 0.9 mL of medium, resulting in the required final drug concentrations. MitoTracker Green (MTG) and LysoTracker green (LTG) (Life Technologies, Rhenium, Jerusalem, Israel) were optimized for concentrations, incubation time and labeling, and added in accordance with the manufacturer’s instructions. LTG was added prior to NPs whereas MTG was added after the addition of NPs and washed for co-localization analysis.

For the study using endocytosis inhibitors, DU-145 cells were pre-incubated with chlorpromazine (20 µM), nystatin (30 µM), chloroquine (100 µM), and indomethacin (300 µM; Sigma Aldrich, St Louis, MO, USA) for 30 min, washed with PBS, followed by the 1-h exposure to 20 µM of (**2**)Ga/HSA NPs at 37 °C. IC_50_ values and optimized concentrations of the inhibitors were assessed in the DU-145 cell line. The final concentration of DMSO (compound required DMSO for solvation) in test culture was <1%.

The interaction of (**2**)Ga/HSA NPs with the plasma membrane were examined using HSA-FITC conjugates are from Sigma (Sigma Aldrich, St Louis, MO, USA). (**2**)Ga/HSA-FITC NPs were formulated in accordance with previously detailed protocols. All cells were incubated in the dark throughout the 4–24 h exposure period and did not receive prolonged exposure to light until excitation by the proper wavelengths. Following 4–24 h of exposure at 37 °C, cellular corrole uptake and organelle labeling was determined using a ×20 objective and a LSM700 confocal system supported with Zen software. Samples were excited at 488 nm for MTG, LTG and HSA-FITC detection (3%, 5%, and 3%, respectively); and at 405 nm (10%) for (**2**)Ga detection.

### Western blot analysis

DU145 cells were seeded in a 6-well plates at a density of 4 × 10^5^ cells/well and allowed to attach and equilibrate overnight. Cells were then pre-treated for 3 h with the vehicle control, 50 µM Z-VAD-FMK or Necrostatin-1, inhibitors of apoptosis or necroptosis, respectively. Following pretreatment, cells were incubated for 20 h with (**2**)Ga/HSA NP (NPs; 5, 10, 15, or 20 µM), NPs (15 µM), or camptothecin (CPT; 5 µM) without the removal of inhibitors. Proteins were isolated from DU145 cells using RIPA lysis and extraction buffer (Thermo Fisher, Waltham, MA) containing cOmplete protease inhibitor cocktail (MilliporeSigma, St. Louis, MO). The amount of protein was quantified using the BCA Protein Assay Kit (Thermo Fisher, Waltham, MA). Protein samples (30 μg) were denatured in Laemmli sample buffer (Bio-rad, Hercules, CA), separated on 4–20% polyacrylamide gradient gel (SDS-PAGE), and transferred onto PVDF membrane using the Trans-Blot Turbo Transfer System (Bio-rad, Hercules, CA). Membranes were probed for the proteins of interest using the following antibodies (each at 1:1000): caspase-3 (#9662), cleaved caspase-3 (#ab32042), PARP (#9542), and β-actin (#4970). Peroxidase-conjugated secondary antibodies against rabbit was used at 1:1000 and immunodetection was achieved using ECL Plus Western Blotting Substrate (Pierce Thermo Fisher, Waltham, MA). Immunoblots were analyzed digitally with a BioRad ChemiDoc Imaging System and quantification was performed using the ImageLab software (Bio-rad, Hercules, CA).

### Live cell IN CELL analysis

Cells (DU-145) were seeded in 24-well microtiter plates with a glass optic bottom (5 × 10^4^ cells/mL; 0.9 mL per well) 24 h before the addition of assigned NPs and compounds. At the time of drug treatment, stock solutions of the compounds were diluted to 10-fold the desired final test concentrations in EMEM medium. Aliquots of 100 μL of these diluted drug solutions were added to the appropriate microtiter wells containing 0.9 mL of medium, resulting in the required final drug concentrations (according to assigned concentration). Fluo-8 AM (Abcam, Zotal LTD, Tel Aviv, Israel) and CellROX green (Life Technologies, Rhenium, Jerusalem, Israel) were added prior to drug treatment in accordance with the manufacturer’s instructions and were optimized for concentrations, incubation time, and labeling results. The final concentration of DMSO (the compound required DMSO for solvation) in test culture was <1%. All cells were incubated in the dark throughout the time of exposure period and did not receive prolonged exposure to light until excitation by the proper wavelengths. Following Fluo-8 AM and CellROX green incubation at 37 °C for 30 min, cells were washed with PBS and fresh medium was added. Plates were taken for fluorescence microscopy imaging using a ×20 (0.95 NA, WD 0.15 mm) objective and a GE IN Cell Analyzer 2000 supported with an IN Cell Miner software for data analysis and image managements. (**2**)Ga was added at the assigned concentrations at the moment of imaging (*t* = 0). For (**2**)Ga uptake imaging and phototoxicity assays, cells were excited using a CFP filter (430_24×) from a white LED light source and emission was recorded using a CY3 filter (579_34×). For both Fluo-8 AM and CellROX green detection, a FITC filter for excitation (490_20×• 500_20×) and emission (525_36m• 535_30m•) was used. Quantification of signals was done using IN Cell software and Microsoft Excel software.

### NPs isolation protocol

Two hundred micromolar (**2**)Ga/HSA NPs were centrifuged at 20,000×*g* for 30 min. The supernatant was transferred to a new tube for two additional centrifugations and transfers. The pellet was resuspended with PBS, pH 7.2, 500 µL and went through two additional cycles of centrifugation and resuspension. After isolation protocols, the concentration of the corrole in each sample (i.e., supernatant and pellet) was assessed using UV-Vis for further in vitro assays.

### Statistical analysis

Data were expressed as mean ± S.E.M and were compared between experimental groups with the use of one-way analysis of variance followed by Tukey’s post hoc test unless otherwise specified (Analyze-it software for Windows Excel, Leeds, UK). Probability values of *p* < 0.05 were considered to be statistically significant.

## Supplementary information

Figure S1

Figure S2

Figure S3

Figure S4

Figure S5

Figure S6

Figure S7

Figure S8

Figure S9

Figure S10

Supplementary Figure Legends

Supplemental Material- Video Legends

Calcium homeostasis perturbation by (2)Ga/HSA NPs.

Calcium homeostasis perturbation by (2)Ga/HSA NPs.

Calcium homeostasis under control treatment.

ROS formation under (2)Ga/HSA NPs treatment.

Video 5

Video 6

Uptake of (2)Ga/HSA NPs by DU-145 cells.

Uptake of (2)Ga/HSA NPs (pellet, isolated by X3 centrifugation protocol) by DU-145 cells.

Uptake of (2)Ga/HSA conjugates (supernatant, isolated by X3 centrifugation protocol) by DU-145 cells.

Extraction assay of (2)Ga/HSA NPs:

HSA role in (2)Ga/HSA NPs uptake by DU-145 cells.

HSA role in (2)Ga/HSA NPs uptake by DU-145 cells, (2)Ga imaging of the same field as in Video 9.

Phototoxicity of (2)Ga/HSA NPs.

Calcium homeostasis under (2)H3/HSA NPs.

Calcium homeostasis under (2)H3/HSA NPs.

Photo toxicity of (2)Ga/HSA NPs.

Photo toxicity of (2)Ga/HSA NPs, control conditions.

Uptake of (2)H3/HSA NPs by DU-145 cells.

Uptake of (2)H3/HSA NPs by DU-145 cells.

HSA role in (2)Ga/HSA NPs uptake by DU-145 cells, HSA-FITC imaging.

HSA role in (2)Ga/HSA NPs uptake by DU-145 cells, (2)Ga imaging in the same field as in Video S8.
