# Supplementary figures and images for: Protein-coated corrole nanoparticles for the treatment of prostate cancer cells
Source: Cell Death Discov. 2020 Jul 28;6:67. doi: 10.1038/s41420-020-0288-x (PMC7387447; doi:10.1038/s41420-020-0288-x)

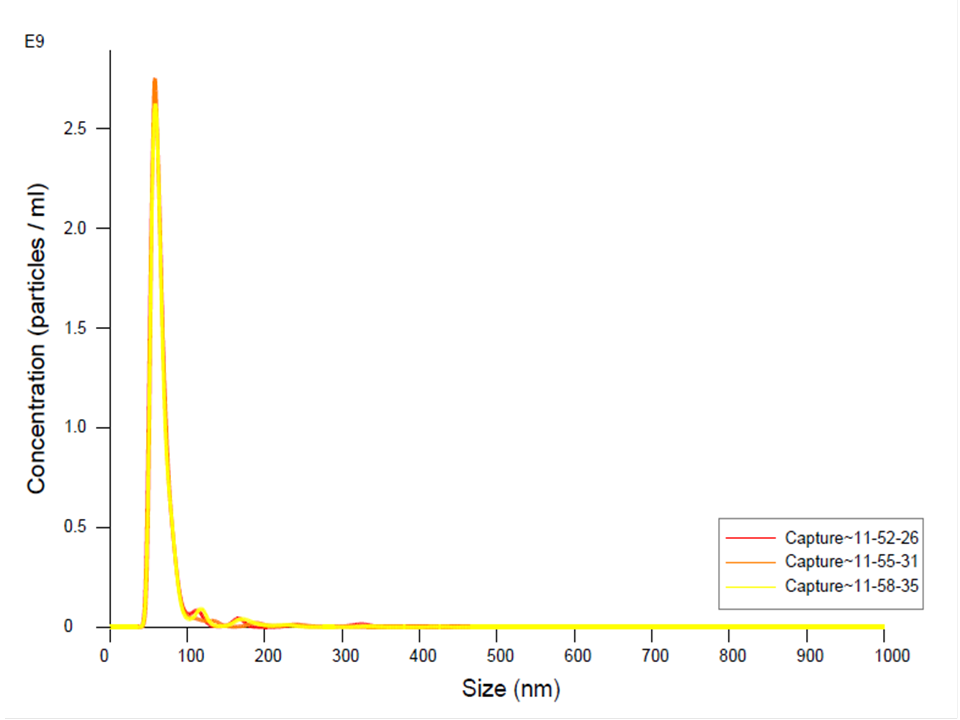

Supplement: Supplementary file 1 — Figure S1 [file 41420_2020_288_MOESM1_ESM.tif]

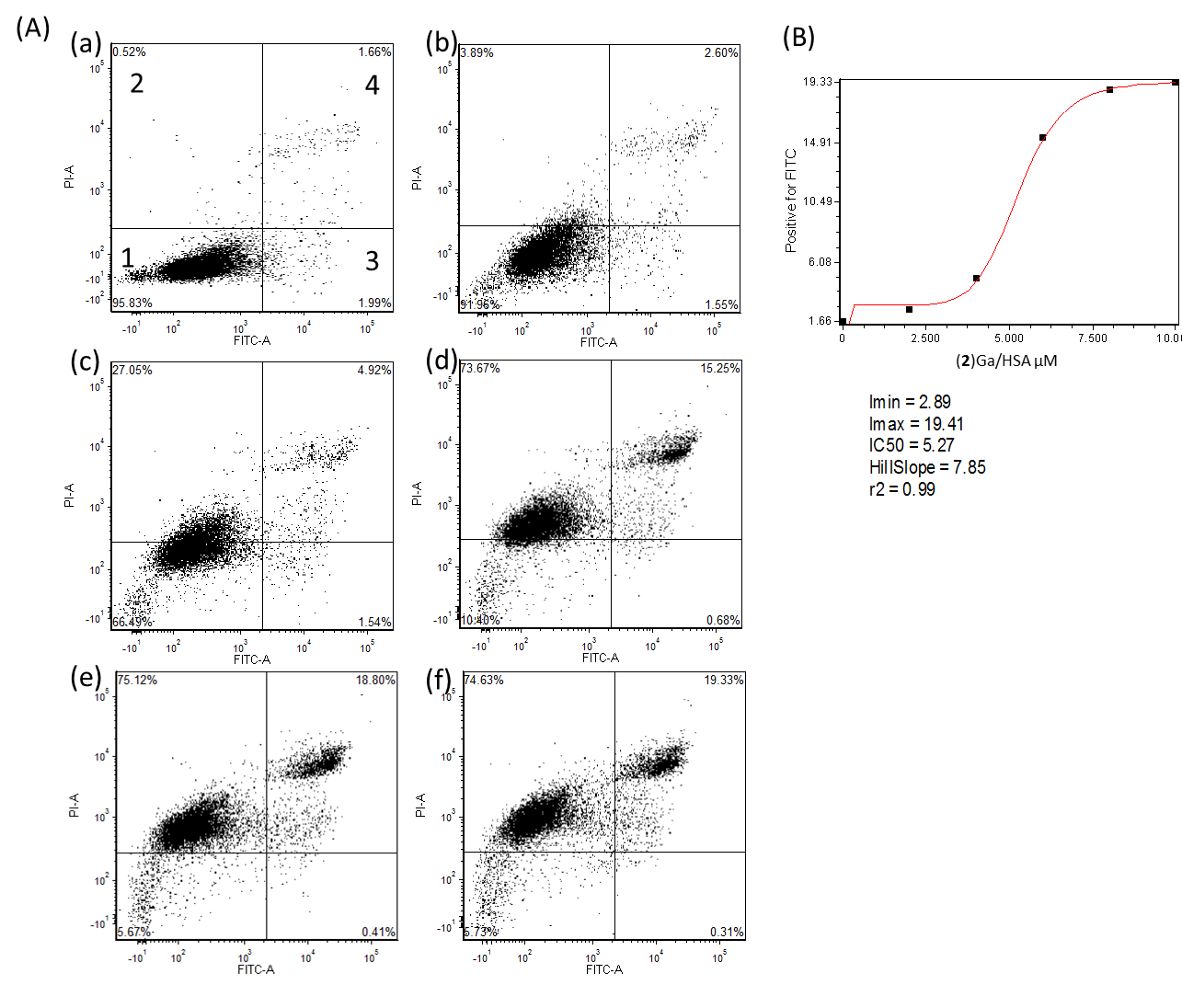

Supplement: Supplementary file 2 — Figure S2 [file 41420_2020_288_MOESM2_ESM.tif]

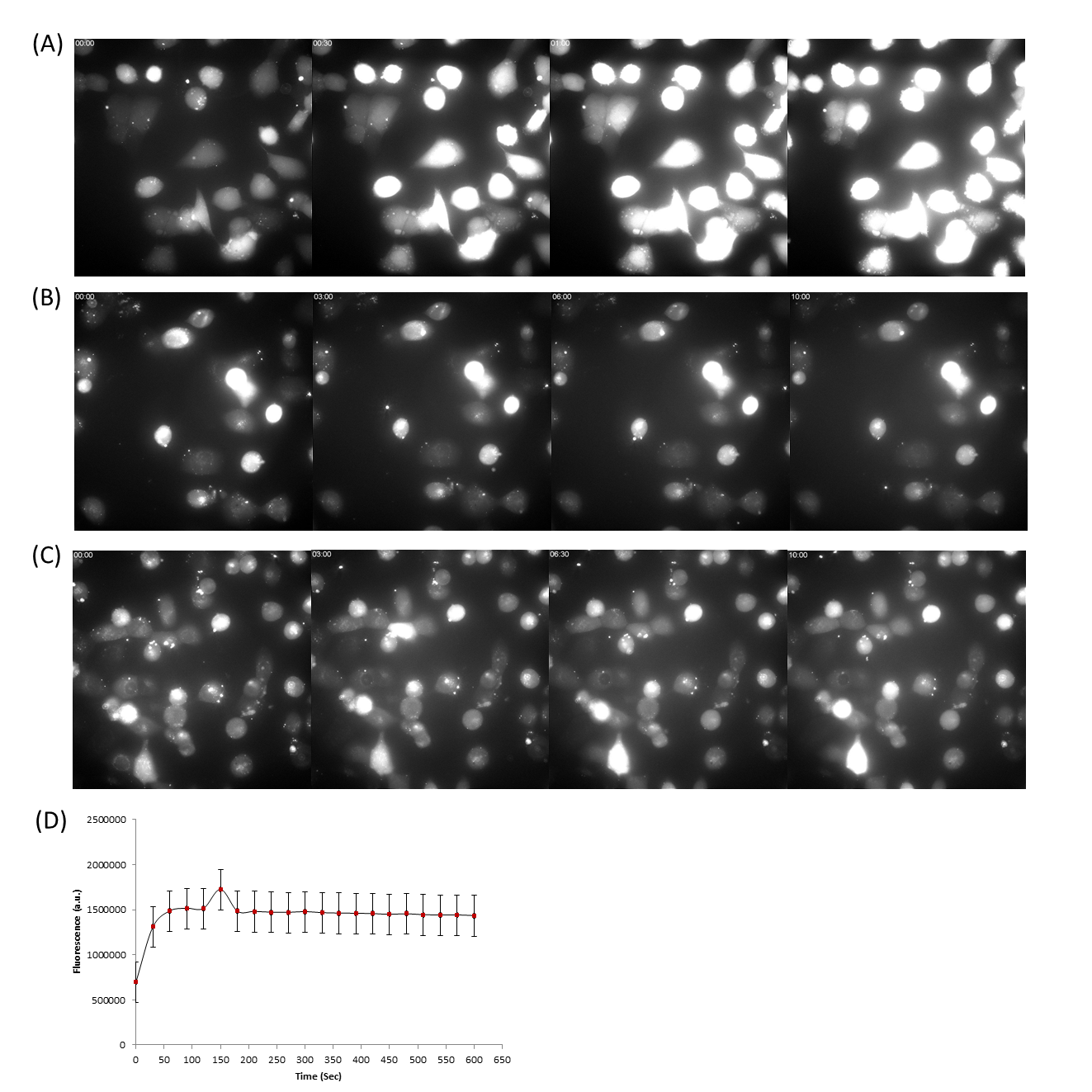

Supplement: Supplementary file 3 — Figure S3 [file 41420_2020_288_MOESM3_ESM.tif]

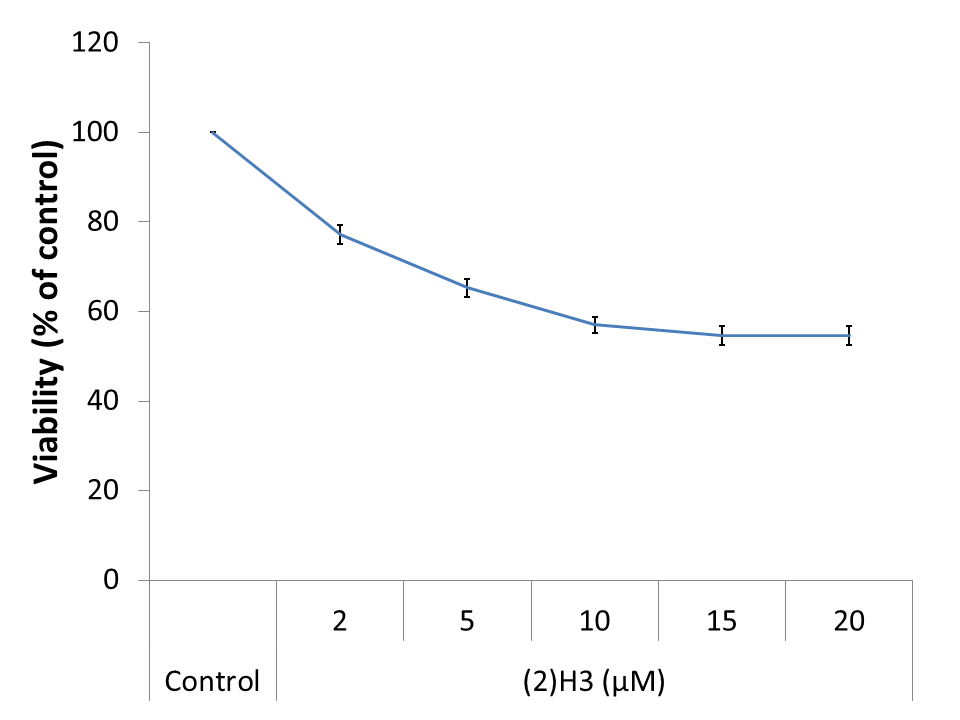

Supplement: Supplementary file 4 — Figure S4 [file 41420_2020_288_MOESM4_ESM.tif]

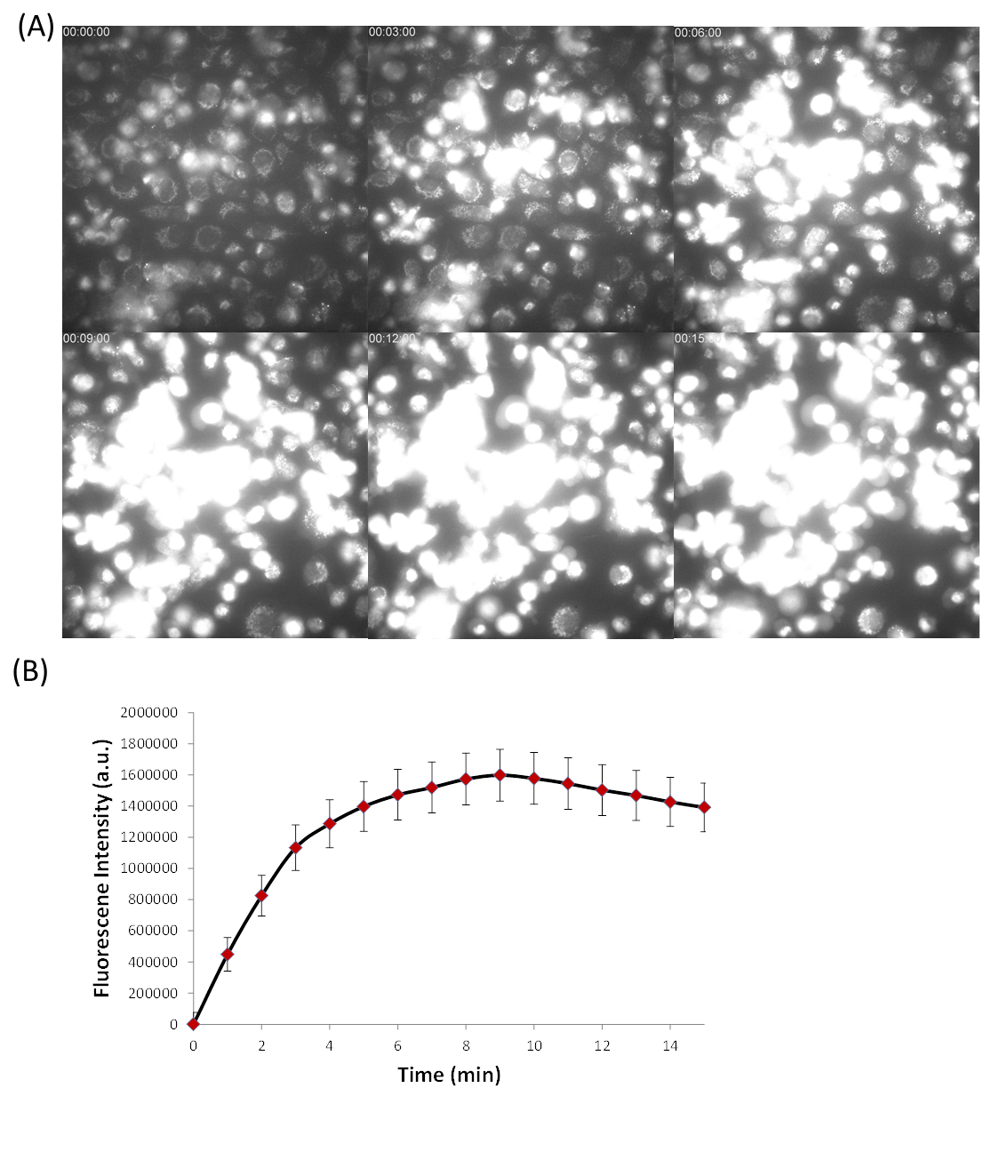

Supplement: Supplementary file 5 — Figure S5 [file 41420_2020_288_MOESM5_ESM.tif]

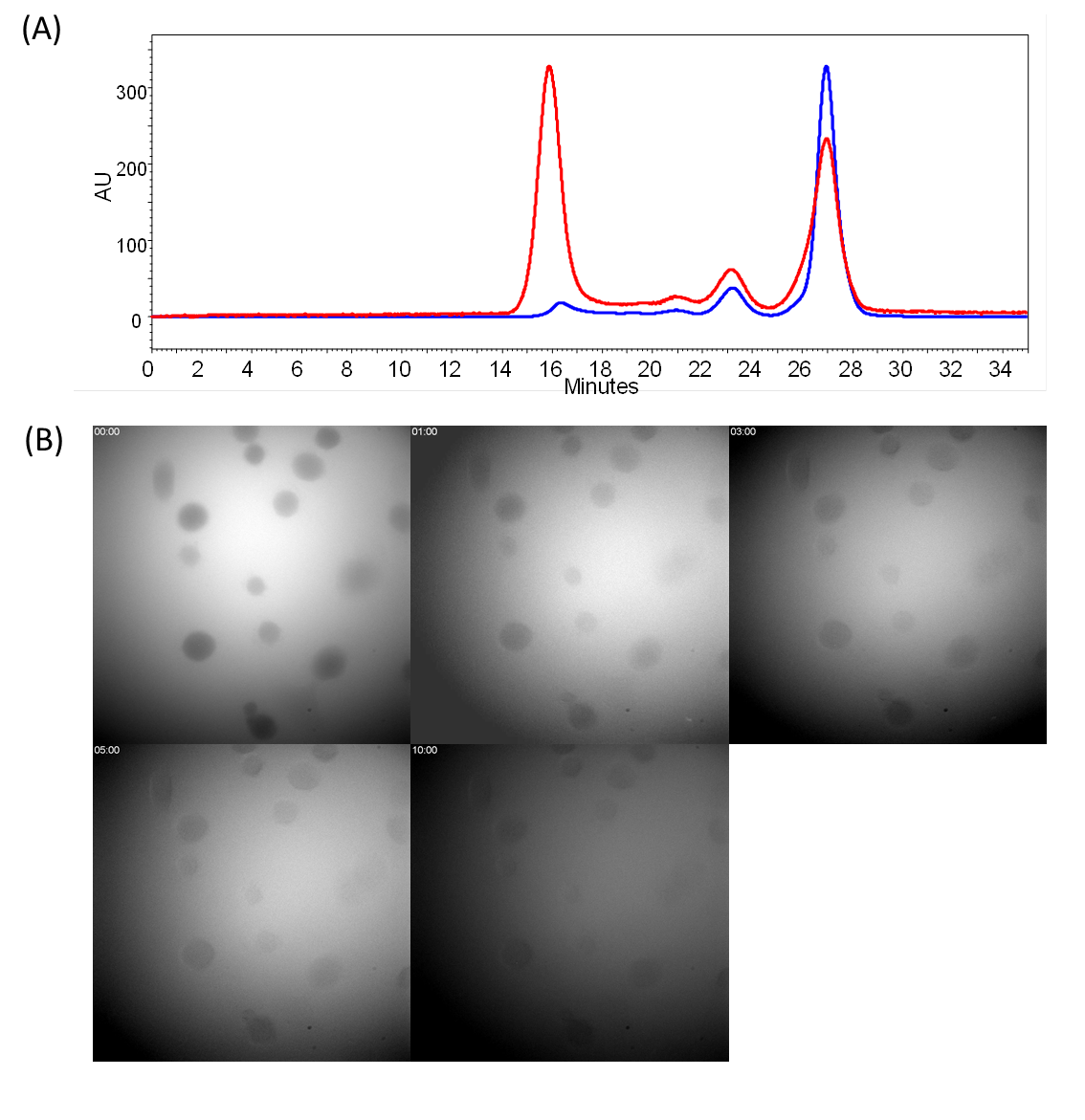

Supplement: Supplementary file 6 — Figure S6 [file 41420_2020_288_MOESM6_ESM.tif]

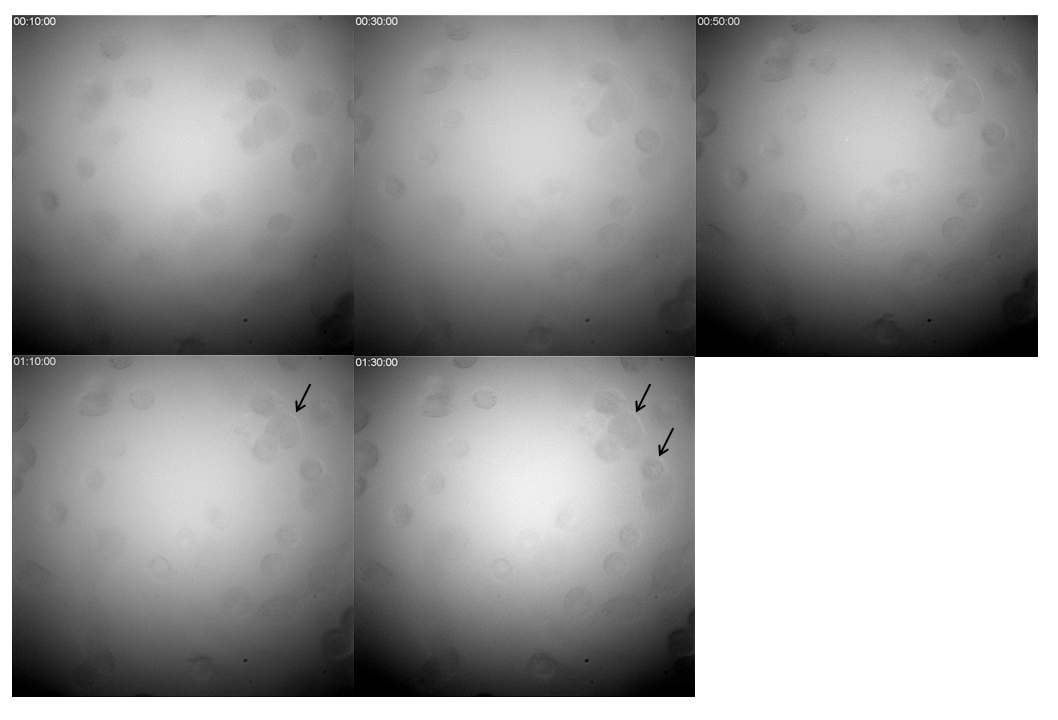

Supplement: Supplementary file 7 — Figure S7 [file 41420_2020_288_MOESM7_ESM.tif]

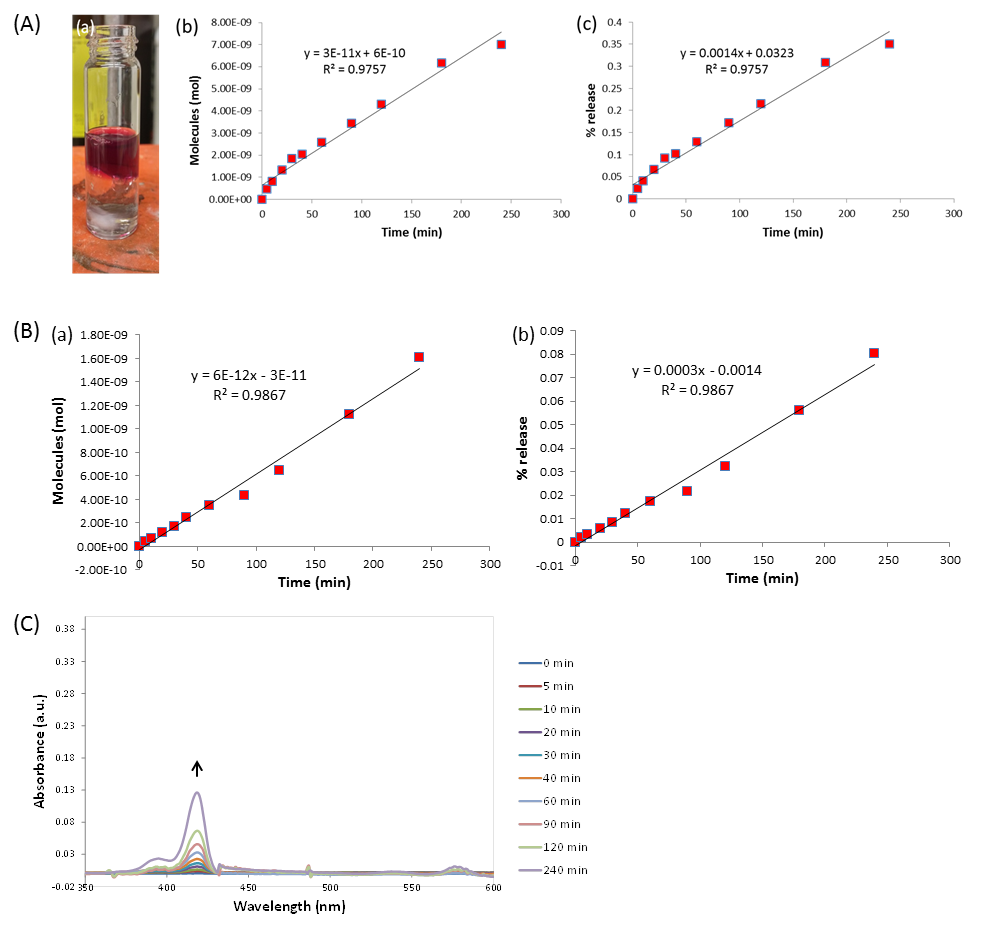

Supplement: Supplementary file 8 — Figure S8 [file 41420_2020_288_MOESM8_ESM.tif]

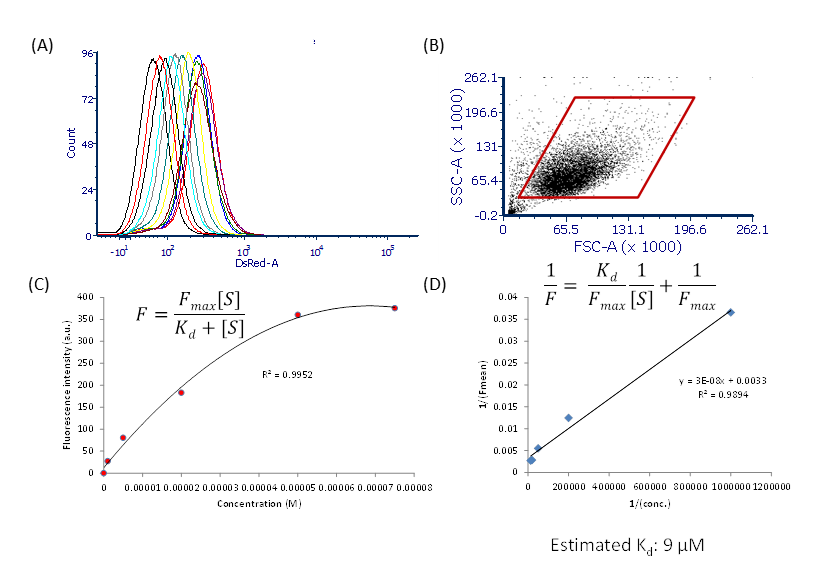

Supplement: Supplementary file 9 — Figure S9 [file 41420_2020_288_MOESM9_ESM.tif]

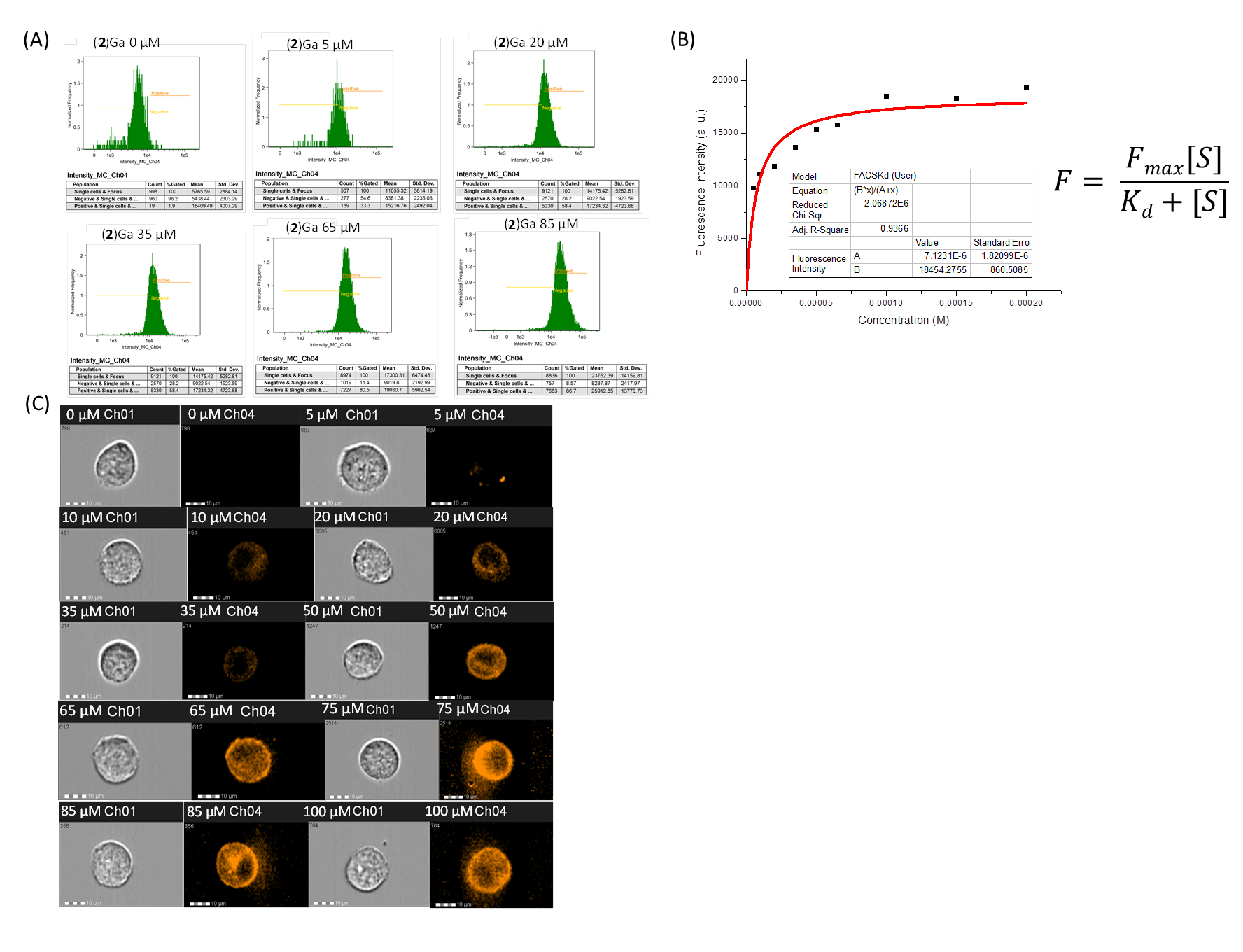

Supplement: Supplementary file 10 — Figure S10 [file 41420_2020_288_MOESM10_ESM.tif]
