# Supplementary material for: Protein-coated corrole nanoparticles for the treatment of prostate cancer cells
Source: Cell Death Discov. 2020 Jul 28;6:67. doi: 10.1038/s41420-020-0288-x (PMC7387447; doi:10.1038/s41420-020-0288-x)
Supplement: Supplementary file 11 — Supplementary Figure Legends [file 41420_2020_288_MOESM11_ESM.docx]

Supporting Information

**Protein-coated corrole-based nanoparticles for the treatment of prostate cancer cells**

*Matan Soll, ^a^ Qiu-Cheng Chen, ^a^ Benny Zhitomirsky, ^b^ Punnajit Lim, ^c^ John Termini, ^ǂc^ Harry B. Gray, ^⯁d^ Yehuda G. Assaraf, ^#b^ and Zeev Gross*^a^*

1. Schulich Faculty of Chemistry, Technion – Israel Institute of Technology, Haifa 32000, Israel
2. The Fred Wyszkowski Cancer Research Laboratory, Dept. of Biology, Technion-Israel Institute of Technology, Haifa 32000003, Israel
3. Department of Molecular Medicine, Beckman Research Institute of the City of Hope, Duarte, CA 91010
4. Beckman Institute, California Institute of Technology, Pasadena, CA 91125

Figure S1 Nanosight NS300 results of (2)Ga/HSA NPs.

Figure S2 (A) FACS analysis of DU-145 prostate cancer cell line treated with an annexin V-FITC kit after 4h incubation with: (a) HSA control; (b)-(f) (2)Ga NPs 2, 5, 10, 15, 20 µM respectively. (B) Plotting of gated cells positive to FITC + PI (i.e., late stage apoptosis) against (2)Ga concentration and assessment of IC_50_ value (IC_50_ = 5.27 µM).

Figure S3 Live time laps imaging of DU-145 cell line (series ranging from time points t= 0, 3, 6 and 10 minutes) incubated with the calcium indicator Fluo-8 AM 4 µM for 30 min prior to washings and the addition of: (A) 2 µM of (2)Ga/HSA particles followed by excitation of (2)Ga at each measured time point, (B) 2 µM of (2)H_3_/HSA particles, (C) 20 µM of (2)H_3_/HSA particles, and (D) quantification of calcium indicator Fluo-8 AM fluorescence obtained from each cell in the 12 separated fields of (A); data points are presented as mean ± SEM n=6. Fluorescence was recorded using a ×40 objective and an IN CELL GE analyzer supported with in cell 2000 software. Samples were excited using a FITC filter for Fuo-8 AM detection and a CFP filter for (2)Ga detection and excitation. Representative images of 12 separate fields, representative results of 3 distinct repeats.

Figure S4 MTT results obtained after 48 hours incubation of DU-145 cell line with increasing concentrations of (2)H3/HSA NPs. IC_50_ values are ambiguous (no value below 50% was obtained). Data points are represented as mean ± SEM n=3, representative results of 3 repeats.

**Figure S5** Live time laps imaging of DU-145 cell line (series ranging from time points t= 0, 3, 6, 9, 12 and 15 minutes) incubated with the ROS indicator CellRox green 10 µM (accumulated mostly in the mitochondria) for 30 min prior to washings and the addition of: (a) 20 µM of (**2**)Ga/HSA NPs followed by excitation of (**2**)Ga at each measured time point, the series of images were taken at time intervals of 1 minutes raging from t= 0 upper left corner to t= 15 min lower right corner, time point is indicated in white on upper left corner of each image; (b) quantification of fluorescence from the center of each cell’s nucleus in the 12 separated fields obtained, data points are presented as mean ± SEM n=6. Fluorescence was recorded using a ×40 objective and an IN CELL GE analyzer supported with in cell 2000 software. Samples were excited using a FITC filter for CellRox green detection and a CFP filter for (4)Ga detection and excitation. Representative images of 12 separate fields, representative results of 3 distinct repeats.

Figure S6 (A) Chromatogram for detection of HSA and the corrole (2)H_3_ in HSA/corrole NPs batch 20092017, with the reading set at 280 nm for detection of HSA (blue line) and at 422 nm for detection of the corrole (red line). NPs displayed slightly larger sizes according to HPLC and DLS: 83.1 ± 25 nm. (B) Live time laps imaging of (2)H_3_/HSA NPs 20 µM uptake into DU-145 cell line: series of images (originally taken at time intervals of 30 sec) raging from t= 0 upper left corner to t= 10 min lower right corner, time point is indicated in white on upper left corner of each image; representative images of chosen time points. Fluorescence was recorded using a ×40 objective and an IN CELL GE analyzer supported with in cell 2000 software. Samples were excited using a CFP filter for (2)H3 detection. Representative images of 12 separate fields, representative results of 3 distinct repeats.

Figure S7 Live time laps imaging of (2)H_3_/HSA NPs 20 µM batch 20092017 (same as figure S6) uptake into DU-145 cell line: series of images (originally taken at time intervals of 10 min) raging from t= 10 min upper left corner to t= 1:30 hours lower right corner, time point is indicated in white on upper left corner of each image; representative images of chosen time points. Fluorescence was recorded using a ×40 objective and an IN CELL GE analyzer supported with in cell 2000 software. Samples were excited using a CFP filter for (2)H_3_ detection. Representative images of 12 separate fields, representative results of 3 distinct repeats. Black arrows indicate cells exhibiting minute fluorescence on the membrane of the cells.

Figure S8 (A) (a) Experimental setup- (2)Ga/HSA dissolved in PBS was stirred below DCM. At any given time points sample were taken to Uv-Vis for the estimation of (2)Ga concentration in DCM using known extinction coefficient. (b) representational graph of molar release rate of (2)Ga. (c) representational graph of release rate of (2)Ga as perscent of original (2)Ga concentration in HSA NPs. Kr (release rate constant) are Kr = 30 pmol/min and Kr = 0.0014 %/min respectively. (B) (a) representational graph of molar release rate of (2)Ga. (c) representational graph of release rate of (2)Ga as perscent of original (2)Ga concentration in HSA NPs. Release rate constants are Kr = 6 pmol/min and Kr = 0.0003 %/min respectively. (C) UV-vis spectra of (2)Ga release into toluene.

Figure S9 (A) FACS analysis of (B) gated cells incubated 10 minutes with elevated concentrations of (2)Ga/HSA NP’s in 4 ^o^C serum free cold EMEM. Cells were immediately pelleted by centrifugation and washed with cold PBS X1 for FACS analysis. Mean fluorescence was plotted against the corresponding concentration; (C) fitted plot to the Langmuir equation (insert); (D) Linearization of the plot and the fitted Langmuir equation yielded an estimation of Kd = 9 ± 1.2 µM. Representative results of three different repeats.

Figure S10 FACS analysis using Amnis ImageStream®X Mark II of cells incubated with elevated concentrations of (2)Ga/HSA NP’s in 4oC and serum free cold EMEM. Cells were immediately taken to the FACS for imaging and analysis (hence the backround fluorescence). Mean fluorescence from gated positive cells (A) were plotted to yield the plot (B) of the Langmuir equation nonlinear fitting. Estmated values of Kd was 7 ± 0.9 µM. (C) Reprasentative images of incubated cells as they are run through the FACS apparatus, both the phase contrast image and the fluorescence and emmision above 600 nm and exitation at 405 nm. Representative results of two different repeats.
