# Supplementary material for: Protein-coated corrole nanoparticles for the treatment of prostate cancer cells
Source: Cell Death Discov. 2020 Jul 28;6:67. doi: 10.1038/s41420-020-0288-x (PMC7387447; doi:10.1038/s41420-020-0288-x)
Supplement: Supplementary file 12 — Supplemental Material- Video Legends [file 41420_2020_288_MOESM12_ESM.docx]

**Video legends**

**Video 1** Calcium homeostasis perturbation by (**2**)Ga/HSA NPs. 10 minutes time-lapse microscopy imaging of live DU-145 cells (using GE IN Cell Analyzer 2000) incubated with the calcium indicator Fluo-8 AM (4 µM) for 30 min prior to triple washes and the addition of 2 µM (2)Ga/HSA NPs. Fluorescence was recorded using a ×20 objective and an IN CELL GE analyzer supported with in cell 2000 software. Samples were excited using a FITC filter for Fluo-8 AM detection [for excitation (490_20x• 500_20x) and emission (525_36m• 535_30m•)]. This video is a representative of 12 separate fields and of three independent experiments.

**Video 2** Calcium homeostasis perturbation by (**2**)Ga/HSA NPs. 10 minutes time-lapse microscopy imaging of live DU-145 cells (using GE IN Cell Analyzer 2000) incubated with the calcium indicator Fluo-8 AM (4 µM) for 30 min prior to triple washes and the addition of 20 µM (2)Ga/HSA NPs. Fluorescence was recorded using a ×20 objective and an IN CELL GE analyzer supported with in cell 2000 software. Samples were excited using a FITC filter for Fluo-8 AM detection [for excitation (490_20x• 500_20x) and emission (525_36m• 535_30m•)]. This video is a representative of 12 separate fields and of three independent experiments.

**Video 3** Calcium homeostasis under control treatment. 10 minutes time-lapse microscopy imaging of live DU-145 cells (using GE IN Cell Analyzer 2000) incubated with the calcium indicator Fluo-8 AM (4 µM) for 30 min prior to triple washes and the addition of HSA. Fluorescence was recorded using a ×20 objective and an IN CELL GE analyzer supported with in cell 2000 software. Samples were excited using a FITC filter for Fluo-8 AM detection [for excitation (490_20x• 500_20x) and emission (525_36m• 535_30m•)]. This video is a representative of 12 separate fields and of three independent experiments.

**Video 4** ROS formation under (**2**)Ga/HSA NPs treatment. 10 minutes time-lapse microscopy imaging of live DU-145 cells (using GE IN Cell Analyzer 2000) incubated with the 10 µM of the ROS indicator CellRox green (accumulated mostly in the nucleus) for 30 min prior to triple washes and the addition of 20 µM of (2)Ga/HSA NPs. Fluorescence was recorded using a ×20 objective and an IN CELL GE analyzer supported with in cell 2000 software. Samples were excited using a FITC filter for CellRox green detection [for excitation (490_20x• 500_20x) and emission (525_36m• 535_30m•)]. This video is a representative of 12 separate fields and of three independent experiments.

**Video 5** Lysosomal structural destabilization by (**2**)Ga/HSA NPs. 30 minutes time-lapse imaging of live DU-145 cells after 30 min of incubation with LysoTracker green (LTG) 150 nM prior to triple washes and the addition of 20 µM (**2**)Ga/HSA NPs. Images were taken using conditions specific for the detection of LTG (green fluorescence). Fluorescence was recorded using a ×20 objective and a LSM700 confocal system microscope supported with Zen software. Samples were excited at 488 nm (3%) for LTG detection. This video is a representative of 12 separate fields and of three independent experiments.

**Video 6** Lysosomal structural destabilization by (**2**)Ga/HSA NPs, (**2**)Ga uptake. 30 minutes time-lapse imaging of live DU-145 cells after 30 min of incubation with LysoTracker green (LTG) 150 nM prior to triple washes and the addition of 20 µM (**2**)Ga/HSA NPs. Images were taken using conditions specific for the detection of (**2**)Ga (red fluorescence), same representative field as in Video 5. Note: some intracellular red auto-fluorescence (might be from LTG) is detectable at t = 0 before the addition of (**2**)Ga/HAS NPs. Fluorescence was recorded using a ×20 objective and a LSM700 confocal system microscope supported with Zen software. Samples were excited at 405 nm (10%) for (**2**)Ga detection. This video is a representative of 12 separate fields and of three independent experiments.

**Video 7** Uptake of (**2**)Ga**/**HSA NPs by DU-145 cells. 10 minutes time-lapse microscopy imaging of live DU-145 cells (using GE IN Cell Analyzer 2000) immediately after the addition of 20 µM of (**2**)Ga/HSA NPs to the well. Fluorescence was recorded using a ×20 objective and an IN CELL GE analyzer supported with in cell 2000 software. For (**2**)Ga imaging cells were excited using a CFP filter (430_24x) and emission was recorded using a CY3 filter (579_34x). This video is a representative of 12 separate fields and of three independent experiments.

**Video 8** Uptake of (**2**)Ga**/**HSA NPs (pellet, isolated by X3 centrifugation protocol) by DU-145 cells. 10 minutes time-lapse microscopy imaging of live DU-145 cells (using GE IN Cell Analyzer 2000) immediately after the addition of 10 µM of (**2**)Ga/HSA NPs to the well. (**2**)Ga**/**HSA NPs were isolated by centrifugation at 5,000 g decanting surfactant and resuspension 3 times bringing the end concentration of corrole to roughly 100 µM using UV-Vis to estimate concentration by absorption at λ_max_ of the soret band. Fluorescence was recorded using a ×20 objective and an IN CELL GE analyzer supported with in cell 2000 software. For (**2**)Ga imaging cells were excited using a CFP filter (430_24x) and emission was recorded using a CY3 filter (579_34x). This video is a representative of 12 separate fields and of three independent experiments.

**Video 9** Uptake of (**2**)Ga**/**HSA conjugates (supernatant, isolated by X3 centrifugation protocol) by DU-145 cells. 10 minutes time-lapse microscopy imaging of live DU-145 cells (using GE IN Cell Analyzer 2000) immediately after the addition of 10 µM of (**2**)Ga/HSA NPs to the well. (**2**)Ga**/**HSA conjugates were isolated by centrifugation at 5,000 g taking surfactant and repeating centrifugation 3 times bringing the end concentration of corrole to roughly 100 µM using UV-Vis to estimate concentration by absorption at λ_max_ of the soret band. Fluorescence was recorded using a ×20 objective and an IN CELL GE analyzer supported with in cell 2000 software. For (**2**)Ga imaging cells were excited using a CFP filter (430_24x) and emission was recorded using a CY3 filter (579_34x). This video is a representative of 12 separate fields and of three independent experiments.

**Video 10** Extraction assay of (**2**)Ga/HSA NPs: 2 ml of 200 µM (**2**)Ga/HSA NPs dissolved in PBS were added to 2 ml DCM and stirred vigorously with DCM for solvation of (**2**)Ga into DCM. Note, no coloration of organic phase could be observed even upon strong stirring. Strongly shaking the sample fails to solvate (**2**)Ga into DCM with a big visible emulsion layer between the two phases.

**Video 11** HSA role in (**2**)Ga/HSA NPs uptake by DU-145 cells. 10 minutes time-lapse microscopy imaging of live DU-145 cells (using GE IN Cell Analyzer 2000) immediately after the addition of 20 µM (**2**)Ga/HSA-FITC NPs. Fluorescence was recorded using a ×10 objective and an IN CELL GE analyzer supported with in cell 2000 software. Samples were excited using a FITC filter for HSA-FITC detection [for excitation (490_20x• 500_20x) and emission (525_36m• 535_30m•)]. This video is a representative of 12 separate fields and of three independent experiments.

**Video 12** HSA role in (**2**)Ga/HSA NPs uptake by DU-145 cells, (**2**)Ga imaging of the same field as in Video 9. 10 minutes time-lapse microscopy imaging of live DU-145 cells (using GE IN Cell Analyzer 2000) immediately after the addition of 20 µM (**2**)Ga/HSA-FITC NPs. Fluorescence was recorded using a ×10 objective and an IN CELL GE analyzer supported with in cell 2000 software. For (**2**)Ga imaging cells were excited using a CFP filter (430_24x) and emission was recorded using a CY3 filter (579_34x). This video is a representative of 12 separate fields and of three independent experiments.

**Video S1** Photo toxicity of (**2**)Ga/HSA NPs. 10 minutes time-lapse microscopy imaging of live DU-145 cells (using GE IN Cell Analyzer 2000) incubated with the calcium indicator Fluo-8 AM (4 µM) for 30 min prior to triple washes and the addition of 20 µM (**2**)Ga/HSA NPs. Fluorescence was recorded using a ×20 objective and an IN CELL GE analyzer supported with in cell 2000 software. Samples were excited using a FITC filter for Fluo-8 AM detection [for excitation (490_20x• 500_20x) and emission (525_36m• 535_30m•)]. For (**2**)Ga excitation and induced photo toxicity cells were excited using a CFP filter (430_24x). This video is a representative of 12 separate fields and of three independent experiments.

**Video S2** Calcium homeostasis under (**2**)H_3_/HSA NPs. 10 minutes time-lapse microscopy imaging of live DU-145 cells (using GE IN Cell Analyzer 2000) incubated with the calcium indicator Fluo-8 AM (4 µM) for 30 min prior to triple washes and the addition of 2 µM (**2**)H_3_/HSA NPs. Fluorescence was recorded using a ×20 objective and an IN CELL GE analyzer supported with in cell 2000 software. Samples were excited using a FITC filter for Fluo-8 AM detection [for excitation (490_20x• 500_20x) and emission (525_36m• 535_30m•)]. This video is a representative of 12 separate fields and of three independent experiments.

**Video S3** Calcium homeostasis under (**2**)H_3_/HSA NPs. 10 minutes time-lapse microscopy imaging of live DU-145 cells (using GE IN Cell Analyzer 2000) incubated with the calcium indicator Fluo-8 AM (4 µM) for 30 min prior to triple washes and the addition of 20 µM (**2**)H_3_/HSA NPs. Fluorescence was recorded using a ×20 objective and an IN CELL GE analyzer supported with in cell 2000 software. Samples were excited using a FITC filter for Fluo-8 AM detection [for excitation (490_20x• 500_20x) and emission (525_36m• 535_30m•)]. This video is a representative of 12 separate fields and of three independent experiments.

**Video S4** Photo toxicity of (**2**)Ga/HAS NPs. 10 minutes time-lapse microscopy imaging of live DU-145 cells (using GE IN Cell Analyzer 2000) incubated with the 10 µM of the ROS indicator CellRox green (accumulated mostly in the nucleus) for 30 min prior to triple washes and the addition of 20 µM of (**2**)Ga/HSA NPs. Fluorescence was recorded using a ×20 objective and an IN CELL GE analyzer supported with in cell 2000 software. Samples were excited using a FITC filter for CellRox green detection [for excitation (490_20x• 500_20x) and emission (525_36m• 535_30m•)]. For (**2**)Ga excitation and induced photo toxicity cells were excited using a CFP filter (430_24x). This video is a representative of 12 separate fields and of three independent experiments.

**Video S5** Photo toxicity of (**2**)Ga/HAS NPs, control conditions. 10 minutes time-lapse microscopy imaging of live DU-145 cells (using GE IN Cell Analyzer 2000) incubated with the 10 µM of the ROS indicator CellRox green (accumulated mostly in the nucleus) for 30 min prior to triple washes and the addition of of HSA alone. Fluorescence was recorded using a ×20 objective and an IN CELL GE analyzer supported with in cell 2000 software. Samples were excited using a FITC filter for CellRox green detection [for excitation (490_20x• 500_20x) and emission (525_36m• 535_30m•)]. For (**2**)Ga excitation and induced photo toxicity cells were excited using a CFP filter (430_24x). This video is a representative of 12 separate fields and of three independent experiments.

**Video S6** Uptake of (**2**)H_3_**/**HSA NPs by DU-145 cells. 10 minutes time-lapse microscopy imaging of live DU-145 cells (using GE IN Cell Analyzer 2000) immediately after the addition of 20 µM of (**2**)H_3_/HSA NPs to the well. Fluorescence was recorded using a ×20 objective and an IN CELL GE analyzer supported with in cell 2000 software. For (**2**)Ga imaging cells were excited using a CFP filter (430_24x) and emission was recorded using a CY3 filter (579_34x). This video is a representative of 12 separate fields and of three independent experiments.

**Video S7** Uptake of (**2**)H_3_**/**HSA NPs by DU-145 cells. 90 minutes time-lapse microscopy imaging of live DU-145 cells (using GE IN Cell Analyzer 2000) immediately after the addition of 20 µM of (**2**)H_3_/HSA NPs to the well. Fluorescence was recorded using a ×20 objective and an IN CELL GE analyzer supported with in cell 2000 software. For (**2**)Ga imaging cells were excited using a CFP filter (430_24x) and emission was recorded using a CY3 filter (579_34x). This video is a representative of 12 separate fields and of three independent experiments.

**Video S8** HSA role in (**2**)Ga/HSA NPs uptake by DU-145 cells, HSA-FITC imaging. 30 minutes time-lapse microscopy imaging of live DU-145 cells immediately after the addition of 20 µM (**2**)Ga/HSA-FITC NPs. Images were taken using conditions specific for the detection of HSA-FITC (green fluorescence). Fluorescence was recorded using a ×20 objective and a LSM700 confocal system microscope supported with Zen software. Samples were excited at 488 nm (5%) for HSA-FITC detection. This video is a representative of 12 separate fields and of three independent experiments.

**Video S9** HSA role in (**2**)Ga/HSA NPs uptake by DU-145 cells, (**2**)Ga imaging in the same field as in Video S8. 30 minutes time-lapse microscopy imaging of live DU-145 cells immediately after the addition of 20 µM (**2**)Ga/HSA-FITC NPs. Images were taken using conditions specific for the detection of (**2**)Ga (red fluorescence). Fluorescence was recorded using a ×20 objective and a LSM700 confocal system microscope supported with Zen software. Samples were excited at 405 nm (10%) for (**2**)Ga detection. This video is a representative of 12 separate fields and of three independent experiments.
